# PSSD: Progressive Spatial-Semantic Decoupling for flow-based gene expression prediction from histology images

**DOI:** 10.1093/bioinformatics/btag598

**Published:** 2026-08-08

**Authors:** Chengyang Zhang, Bo Li, Bob Zhang, Yuansong Zeng, Yuhao Yi, Jiancheng Lv

**Affiliations:** College of Computer Science, Sichuan University, Chengdu, China; Department of Pathology and Institute of Clinical Pathology, West China Hospital, Sichuan University, Chengdu, China; Department of Computer and Information Science, University of Macau, Macau, China; Department of Computer and Information Science, University of Macau, Macau, China; School of Big Data and Software Engineering, Chongqing University, Chongqing, China; College of Computer Science, Sichuan University, Chengdu, China; Department of Pathology and Institute of Clinical Pathology, West China Hospital, Sichuan University, Chengdu, China; College of Computer Science, Sichuan University, Chengdu, China

## Abstract

**Motivation:**

Predicting spatial gene expression from histology images offers a cost-effective complement to spatial transcriptomics. However, existing methods struggle to balance spatial continuity with functional heterogeneity, often producing over-smoothed predictions or neglecting spatial context.

**Results:**

We present PSSD, a conditional flow matching framework with progressive spatial-semantic decoupling. PSSD models spatial and semantic information through separate but interacting pathways and employs a three-stage architecture with decoupled flows, adaptive fusion, and cross-stream coupling to generate biologically coherent, high-fidelity gene expression profiles. Across seven spatial transcriptomics datasets spanning multiple tissues and resolutions, PSSD consistently achieved the highest Pearson correlation coefficients among the compared methods while better preserving biological boundaries and spatial autocorrelation. Under the same sampling protocol, PSSD reduced inference time from 32.04 to 3.98 min per sample on DLPFC compared with the diffusion-based Stem model and achieved approximately sevenfold acceleration on the BC and cSCC datasets without compromising predictive quality. These results demonstrate that flow-based spatial-semantic decoupling provides an effective and computationally efficient bridge between histology and transcriptomics.

**Availability and implementation:**

The source code and data are available at https://github.com/ChyaZhang/PSSD.

## 1 Introduction

In recent years, computer vision has achieved remarkable success in computational pathology, transforming our ability to extract structural and morphological information from histological slides. The next frontier lies in decoding the underlying molecular landscape directly from visual tissue patterns. Among these emerging directions, predicting spatial gene expression directly from routine Hematoxylin and Eosin (H&E)-stained histology images has drawn growing attention (Williams *et al.* 2022, Schott *et al.* 2024). This task offers a promising and cost-effective alternative to Spatial Transcriptomics (ST) experiments, which remain expensive and technically demanding (Ståhl *et al.* 2016, Poovathingal *et al.* 2024). Leveraging the abundance of routinely collected H&E slides, this approach holds potential to uncover molecular insights into tissue microenvironments, disease progression, and therapeutic response at scale (Nguyen *et al.* 2017, Rana *et al.* 2020).

Despite significant progress, existing methods for this task face a key challenge: jointly modeling spatial continuity and functional heterogeneity in tissue architecture. Spatial continuity refers to neighboring regions having correlated expression, whereas biological function is heterogeneous. Adjacent spots may belong to distinct microenvironments, such as tumors or immune stroma, that have drastically different gene profiles. Current methods struggle to balance these aspects: a unified representation can lead to over-smoothing and loss of biological specificity, while ignoring spatial structure can compromise contextual coherence.

Early efforts, such as ST-Net (He *et al.* 2020), HisToGene (Pang *et al.* 2021), and Hist2ST (Zeng *et al.* 2022), formulate histology-to-expression prediction as a regression problem that directly maps image patches to expression profiles. These methods typically encode spatial coordinates and visual features into a shared latent space, which inadvertently enforces local smoothness at the cost of biological boundaries. As a result, predictions in heterogeneous regions tend to be oversimplified, blurring the fine-grained molecular distinctions crucial for downstream interpretation. Recently, generative modeling has emerged as a more powerful paradigm for this task. For instance, Stem (Zhu *et al.* 2025) formulates expression reconstruction as a conditional denoising process, improving fidelity and diversity of predicted profiles. However, Stem does not explicitly incorporate spatial information, failing to disentangle spatial and functional factors. Moreover, its iterative sampling procedures impose heavy computational costs, limiting scalability to whole-slide applications. Although subsequent flow-based methods such as STFlow (Huang *et al.* 2025) achieve faster sampling through flow matching, they still rely on local neighborhood aggregation, which conflates spatial dependence with functional heterogeneity.

To address this challenge, we argue that resolving the spatial-functional trade-off requires moving beyond static feature-level compromise. Prior strategies rely on rigid architectural branches that force a static aggregation, resulting in either over-smoothing or loss of spatial coherence. In this work, we propose PSSD (Progressive Spatial-Semantic Decoupling), which formulates spatial-semantic decoupling as a dynamic trajectory optimization problem within a conditional flow matching framework. Instead of merely combining modalities at the network level, our core insight is to embed the decoupling mechanism directly into the continuous vector field. This paradigm shift enables the generative process to engage in a stepwise, adaptive interaction along the Ordinary Differential Equation (ODE) integration, suppressing spatial priors in functionally heterogeneous regions while reinforcing them in structurally coherent domains. Consequently, flow matching is not merely adopted for sampling efficiency, but serves as the essential continuous mathematical substrate that makes this trajectory-guided decoupling possible. We evaluate PSSD on multiple publicly available ST datasets. The experimental results indicate that PSSD consistently outperforms state-of-the-art methods, positioning the flow-based decoupling strategy as a more natural framework for bridging histology and transcriptomics. The main contributions of this study are summarized as follows:

- We introduce the progressive spatial–semantic decoupling within a continuous flow matching framework. Unlike traditional dual-branch architectures that force a rigid feature compromise, PSSD enables a trade-off between spatial continuity and functional heterogeneity.
- We design a gated coupling mechanism that prevents the complete separation of modalities. By evaluating spatial variance along the continuous vector field, the model adaptively modulates the integration trajectory.
- We conduct comprehensive experiments on multiple ST datasets, demonstrating that PSSD achieves SOTA performance across key metrics and generates biologically meaningful spatial transcriptomic profiles.

## 2 Methods

**Figure 1:**
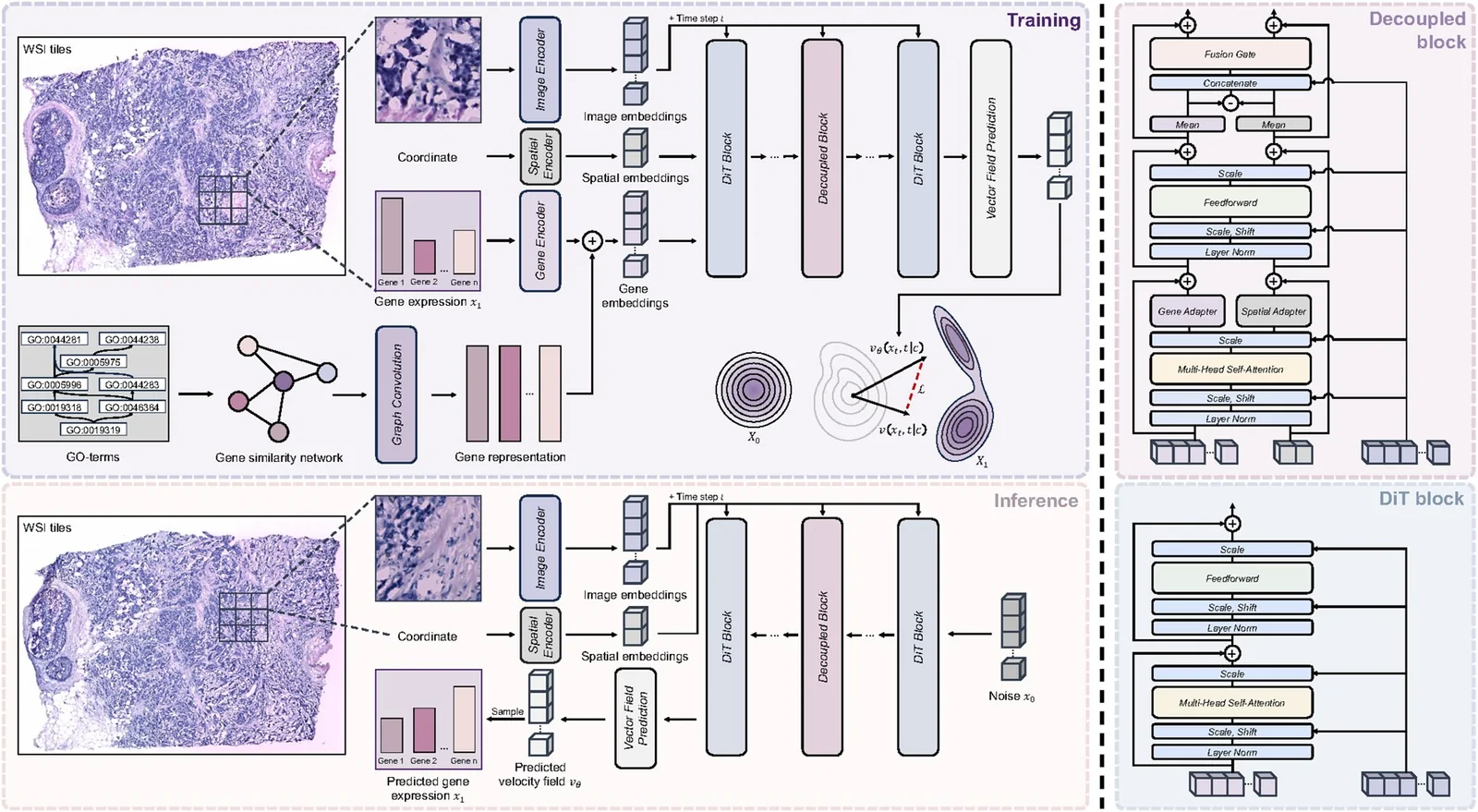
Overview of the proposed PSSD. The training input of PSSD contains H&E images, Gene Ontology (GO)-enhanced gene representations, spatial coordinates, and spot-wise gene expression profiles. During training, gene profiles and gene representations are separately embedded and combined as inputs into the network, together with embedded spatial coordinates. H&E images are cropped into patches centered on each spot and tokenized using pathology foundation models. The image tokens serve as conditioning information, which is injected into every DiT block and decoupled block. During inference, PSSD samples gene expression predictions from random distributions conditioned on any input image patch.

In this section, we present PSSD in detail, a progressive spatial-semantic decoupling framework based on flow matching for accurate gene expression prediction from histology images. An overview of PSSD is provided in Fig. 1.

### 2.1 Preliminaries

Flow matching (Albergo and Vanden-Eijnden 2022, Lipman *et al.* 2022) is a generative modeling framework that formulates data synthesis as learning a continuous flow from a simple base distribution to the target data distribution. Unlike standard diffusion models, which require a discretized forward-noise process, flow matching directly learns a vector field that deterministically transports samples from a known prior to the target data distribution.

Formally, let $x_{0}∼p_{0}(x)$ denote a sample from the prior data distribution (e.g. Gaussian), and $x_{1}∼p_{1}(x)$ denote a sample from the target distribution. Flow matching defines a time-dependent vector field $v_{\theta }(x,t)$, parameterized by a neural network θ, to represent the instantaneous direction and magnitude of change at each point. This vector field is governed by the following ODE:


(1) $$\frac{dx_{t}}{dt}=v_{\theta }(x_{t},t),\;t\in [0,1].$$

The training objective is derived by aligning the model’s instantaneous velocity with a target transport direction. Following the rectified flow formulation (Liu *et al.* 2022), we apply linear interpolation as the transport path:


(2) $$x_{t}=(1-t)x_{0}+tx_{1}.$$

Along this linear path, the velocity field is constant and given by $v_{\theta }(x_{t},t)=x_{1}-x_{0}$, representing the optimal transport direction at each point. To approximate this optimal velocity field, the neural network θ is optimized by minimizing:


(3) $$L=E_{x_{0}∼p_{0},x_{1}∼p_{1},t∼u[0,1]}{\left\|v_{\theta }\left(x_{t},t\right)-v\left(x_{t},t\right)\right\|}_{2}^{2}.$$

After training, generation is performed by sampling $x_{0}$ and numerically integrating the learned ODE from t=0 to t=1. Formally, the generated sample $x_{1}$ is obtained as:


(4) $$x_{1}=x_{0}+\int_{0}^{1}v_{\theta }(x_{t},t)dt,$$

which approximates a sample from $p_{1}$. In practice, standard ODE solvers, such as Euler and Runge-Kutta, are used to discretize the integral.

### 2.2 Problem formulation

Let *N* denote the number of spatial spots, and $S\in R^{N\times d}$ denote their embedded spatial coordinates, where *d* is the hidden dimension. For each spot, an associated histology patch is extracted and encoded into an image embedding $z\in R^{d}$. The goal is to predict the gene expression profile $X\in R^{N\times G}$, where *G* is the number of genes.

Traditional methods often treat this task as a regression problem, which assumes a one-to-one mapping between inputs and gene expression profiles. Considering the inherent uncertainties in tissue heterogeneity and the stochastic nature of gene expression, this paradigm may fail to capture the multimodal distribution of possible expression patterns. To address this limitation, we reformulate the problem as learning the conditional distribution of gene expression given spatial and image information:


(5) $$x∼p_{\text{gene}}(x|s,z).$$

To model the conditional distribution, we extend flow matching by introducing spatial coordinates and image embeddings as conditioning variables c=(s,z). The source distribution $p_{0}$ represents prior samples of gene expression, while the target distribution becomes condition-dependent, denoted as $p_{1}(x|c)$. Our goal is to learn a conditional vector field $v_{\theta }$ that captures condition-specific transformations:


(6) $$\frac{d}{dt}x_{t}=v_{\theta }(x_{t},t|c),\;x_{0}∼p_{0},\;x_{1}∼p_{1}.$$

### 2.3 Image embedding extraction

For each spot in the ST data, we leverage pathology foundation models to extract embeddings of the corresponding histology image patches. These models are designed to capture general histological patterns and have demonstrated strong transferability across datasets and tasks (Ciga *et al.* 2022, Xu *et al.* 2024, Guo *et al.* 2025). Specifically, we employ two state-of-the-art encoders, UNI (Chen *et al.* 2024) and CONCH (Lu *et al.* 2024), both pretrained on large-scale whole-slide image datasets. While using a single encoder already yields competitive results, experiments in Appendix D suggest that combining UNI and CONCH provides complementary benefits, resulting in substantial improvements within the generative modeling framework.

To obtain a compact latent embedding that aggregates patch-level information, we apply attention pooling to the output embeddings, producing a single representation. This representation is further mapped to the hidden dimension of PSSD via a Multi-Layer Perceptron (MLP). Finally, given a set of image patches, we obtain a sequence of latent embeddings $\left\{z^{1},\ldots ,z^{n}\right\}$, which inject histological context into the generative prediction of gene expression profiles.

### 2.4 Gene embedding construction

To capture both quantitative and relational characteristics of genes, we construct a joint gene embedding *h* that integrates gene expression counts and gene–gene correlations:


(7) $$h=h^{\text{count}}+h^{\text{corr}}.$$

Expression counts provide direct information on transcriptional activity across spatial locations, whereas gene–gene correlations reflect functional relationships derived from prior biological knowledge. By combining these two sources, the joint embedding preserves both the individual gene activity and the structural dependencies. For each gene, expression counts are first transformed into a continuous representation. Specifically, given an expression vector $x\in R^{G}$, the count embedding $h^{\text{count}}\in R^{G\times d}$ is computed by passing *x* through an MLP.

To incorporate the biological prior, we enrich the embedding space with gene–gene correlations derived from external knowledge. We leverage pretrained embedding *E* from Gene2Vec (Du *et al.* 2019), further enhanced with the Gene Ontology (GO) graph, which encodes functional similarity among genes. The GO provides a structured vocabulary organized into three domains: biological process, molecular function, and cellular component. Each domain is represented as a Directed Acyclic Graph (DAG), where edges denote parent–child relations between terms. Each GO term is first assigned an Information Content (IC) value to quantify its specificity:


(8) IC(GO)=−log(freq(GO)),

where freq(GO) denotes the frequency of annotations involving term GO. Given two terms ${\text{GO}}_{a}$ and ${\text{GO}}_{b}$ within the same domain, we identify their Lowest Common Ancestor (LCA) with the maximum IC and compute similarity as:


(9) $$\text{Sim}({\text{GO}}_{a},{\text{GO}}_{b})=\frac{2\times \text{IC}(\text{LCA}({\text{GO}}_{a},{\text{GO}}_{b}))}{\text{IC}({\text{GO}}_{a})+\text{IC}({\text{GO}}_{b})}.$$

This score captures shared information between terms and is set to zero when they belong to different domains.

For genes $g_{i}$ and $g_{j}$ annotated with $n_{i}$ and $n_{j}$ different GO terms, we construct a similarity matrix $M\in R^{n_{i}\times n_{j}}$, where each entry quantifies the similarity between two GO terms: $M_{m,k}=\text{Sim}({\text{GO}}_{m}^{i},{\text{GO}}_{k}^{j})$, with ${\text{GO}}_{m}^{i}$ and ${\text{GO}}_{k}^{j}$ denoting the *m*-th and *k*-th GO terms associated with genes $g_{i}$ and $g_{j}$. To obtain a robust gene similarity score, we apply the Hungarian algorithm to find an optimal one-to-one alignment between the two sets of terms. Let the resulting optimal matching M(i,j) defines a set of aligned index pairs, the gene similarity is then defined as:


(10) $$\text{Sim}(g_{i},g_{j})=\frac{1}{\text{min}(n_{i},n_{j})}\sum_{\left(r,w)\in M(i,j\right)}M_{r,w}.$$

Repeating this process across all gene pairs yields a similarity matrix $A\in R^{G\times G}$. For each gene $g_{i}$, we compute a threshold $\theta_{i}$ as the mean similarity over its top ten neighbors and retain an edge $\left(g_{i},g_{j}\right)$ if $A_{ij}>\theta_{i}$. This results in a weighted gene graph $G_{g}=(V,E)$, where edge weights correspond to the computed similarities. Finally, given pretrained embeddings *E*, we employ a Graph Neural Network (GNN) defined on $G_{g}$ to project them into the hidden dimension:


(11) $$h^{\text{corr}}=\text{GNN}(E,G_{g})\in R^{G\times d}.$$

The GNN aggregates relational information along the constructed graph, ensuring that the resulting embeddings capture both prior biological knowledge and functional dependencies among genes.

To keep the two streams comparable in scale before summation, raw expression counts are preprocessed following the HEST-1k benchmark protocol (Jaume *et al.* 2024a) (per-spot total-count normalization followed by log(1+·) transformation), while the Gene2Vec embeddings used to build $h^{\text{corr}}$ are unit-scale pretrained vectors. The MLP and GNN share the same hidden dimension and standard initialization, and the LayerNorm at the entry of each DiT block further re-normalizes the joint embedding, preventing either modality from dominating.

### 2.5 Progressive decoupling network

To address the inherent tension in ST data between spatial continuity and functional heterogeneity, we design a progressive decoupling network that explicitly decouples spatial and semantic representations. By embedding the decoupling mechanism within the continuous vector field of flow matching, PSSD transforms structural disentanglement into a dynamic optimization process. As the state variable evolves along the ODE integration path from noise to data, the network adaptively reconciles conflicting spatial and semantic signals. This network employs a three-stage progressive architecture that gradually transitions from shared to decoupled to fused representations.

#### 2.5.1 Shared encoding

The initial blocks process the joint gene embedding *h* through standard DiT (Peebles and Xie 2023) blocks with temporal and image conditions:


(12) $$h^{\prime }=\text{DiTBlocks}(h,c),\;c=t+z,$$

where *t* and *z* represent time and image embeddings, respectively. DiT is initially developed for class-conditional image generation, employing a modulation mechanism that propagates the influence of diffusion timesteps and input conditions throughout all layers. For generating conditional gene expression, we modify DiT by removing image-specific modules, such as the patchifier and unpatchifier. This stage captures modality-invariant semantics while remaining agnostic to spatial structure.

#### 2.5.2 Spatial-semantic decoupling

In this stage, $h^{\prime }$ is decoupled into a semantic flow $h^{\text{se}}$ and a spatial flow $h^{\text{sp}}$. The semantic flow preserves genetic information, while the spatial flow incorporates coordinate modulation. Specifically, we employ normalized spot-wise embedding (Min-Max scaled + MLP) to encode spot coordinates as $s\in R^{G\times d}$, and initialize the two flows as:


(13) $$h^{\text{sp}}=h^{\prime }+\alpha \cdot s,\;h^{\text{se}}=h^{\prime },$$

where α is a scaling factor that prevents spatial bias from dominating expression features. Both flows are then processed through shared attention and feedforward modules, but each employs specific adapters through lightweight linear transformations:


(14) $$\begin{array}{c} {\tilde{h}}^{\text{sp}}=h^{\text{sp}}+{\text{Adapter}}^{\text{sp}}(\text{Attn}(\text{LN}(h^{\text{sp}}))),\\ {\tilde{h}}^{\text{se}}=h^{\text{se}}+{\text{Adapter}}^{\text{se}}(\text{Attn}(\text{LN}(h^{\text{se}}))). \end{array}$$

To enable selective interaction between the two flows, we introduce a gating mechanism that determines their cross update:


(15) $$\phi =\text{Softmax}(\text{MLP}([c,{\bar{h}}^{\text{sp}},{\bar{h}}^{\text{se}},\left|{\bar{h}}^{\text{sp}}-{\bar{h}}^{\text{se}}\right|])),$$

(16) $${\bar{h}}^{\text{sp}}\leftarrow {\bar{h}}^{\text{sp}}+\phi_{1}\cdot {\bar{h}}^{\text{se}},\;{\bar{h}}^{\text{se}}\leftarrow {\bar{h}}^{\text{se}}+\phi_{2}\cdot {\bar{h}}^{\text{sp}},$$

where $\bar{h}$ is the mean value of $\tilde{h}+\text{FFN}(\text{LN}(\tilde{h}))$. This bidirectional coupling allows controlled information exchange while preserving the identity of each flow. The progressive nature of this exchange across multiple blocks enables the model to gradually refine the balance between spatial coherence and semantic accuracy.

#### 2.5.3 Adaptive fusion

In the final fusion stage, the two flows are reconciled through adaptive spot-wise weighting. For each spot we compute a fusion coefficient $\lambda =\sigma (f_{\text{fusion}}(s))$, with σ the sigmoid function and $f_{\text{fusion}}$ a lightweight projection of the coordinates. The fused representation is then formed as:


(17) $$h^{\text{fuse}}=\lambda {\bar{h}}^{\text{sp}}+(1-\lambda ){\bar{h}}^{\text{se}},$$

which adaptively balances spatial and semantic contributions at each location. The fused embedding is further refined through additional DiT blocks before generating the final gene expression predictions. By progressively integrating the two flows rather than relying on naive neighborhood aggregation, this stage preserves local functional heterogeneity while still exploiting spatial dependencies when beneficial.

## 3 Experiments

### 3.1 Experimental setup

#### 3.1.1 Datasets

We evaluate the proposed PSSD on three ST datasets from the HEST-1k benchmark (Jaume *et al.* 2024a): DLPFC (Maynard *et al.* 2021), BC (Andersson *et al.* 2020), and cSCC (Ji *et al.* 2020), which are evaluated in (Xie *et al.* 2023). Specifically, the DLPFC dataset contains 12 brain tissue slices from three individuals, with each individual contributing four slices. The number of spatial spots per slice ranges from 3460 to 4789, covering a total of 33 538 genes. The BC dataset provides a breast cancer atlas consisting of 12 slices with 3777 spots in total. Additionally, the cSCC dataset comprises 12 human skin squamous cell carcinoma slices, each containing 521 to 1182 spots. To further assess generalization across additional tissue types and modern spatial transcriptomics platforms, we extend the evaluation to three additional HEST-1k Visium datasets (ccRCC, PAAD, and COAD) covering kidney, pancreas, and bowel tissues, as well as one higher-resolution Visium HD COAD dataset that captures fine-scale tissue architecture beyond standard Visium. Detailed information for each slice is provided in Tables A.1–A.3 of Appendix A, available as supplementary data at *Bioinformatics* online.

**Table 1:** Quantitative comparison of PSSD and baseline methods on three ST datasets.

Datasets	**DLPFC**		**BC**		**cSCC**	
Methods	HVG	HEG	HVG	HEG	HVG	HEG
Hist2ST (Zeng *et al.* 2022)	0.109 ± 0.0691	0.052 ± 0.0824	0.093 ± 0.0821	0.085 ± 0.1465	0.097 ± 0.0540	0.081 ± 0.0026
HisToGene (Pang *et al.* 2021)	0.116 ± 0.0274	0.063 ± 0.0607	0.102 ± 0.0805	0.091 ± 0.0184	0.121 ± 0.0059	0.062 ± 0.1843
IHItoGene (Jia *et al.* 2023)	0.124 ± 0.0523	0.095 ± 0.1319	0.191 ± 0.0644	0.118 ± 0.0514	0.143 ± 0.1832	0.158 ± 0.0175
ST-Net (He *et al.* 2020)	0.210 ± 0.0127	0.117 ± 0.0555	0.219 ± 0.0582	0.207 ± 0.1009	0.105 ± 0.0557	0.113 ± 0.0171
TRIPLEX (Chung *et al.* 2024)	0.236 ± 0.0230	0.154 ± 0.0898	0.246 ± 0.0402	0.205 ± 0.0428	0.251 ± 0.0934	0.229 ± 0.0592
TANGLE (Jaume *et al.* 2024b)	0.241 ± 0.0209	0.186 ± 0.0932	0.254 ± 0.0327	0.149 ± 0.0936	0.263 ± 0.0601	0.174 ± 0.0977
BLEEP (Xie *et al.* 2023)	0.252 ± 0.0617	0.173 ± 0.0920	0.227 ± 0.0775	0.223 ± 0.0344	0.254 ± 0.0574	0.209 ± 0.0312
M2TGLGO (Shi *et al.* 2025)	0.291 ± 0.0218	0.217 ± 0.0431	0.308 ± 0.0501	0.269 ± 0.0409	0.296 ± 0.0433	0.256 ± 0.0489
Stem (Zhu *et al.* 2025)	0.300 ± 0.0921	0.267 ± 0.0745	0.348 ± 0.0578	0.351 ± 0.0549	0.368 ± 0.2099	0.362 ± 0.2112
STFlow (Huang *et al.* 2025)	0.313 ± 0.0872	0.279 ± 0.0795	0.361 ± 0.0647	0.358 ± 0.0610	0.383 ± 0.1975	0.390 ± 0.1937
PSSD (Ours)	**0.337** ± **0.0770**	**0.299** ± **0.0673**	**0.369** ± **0.0613**	**0.370** ± **0.0591**	**0.416** ± **0.1837**	**0.411** ± **0.1856**

Reported values are Pearson Correlation Coefficients (PCC↑) for the top 50 Highly Variable Genes (HVGs) and top 50 Highly Expressed Genes (HEGs). The best results are in **bold**; the second-best results are underlined.

#### 3.1.2 Implementation details

In the training stage, we employ the leave-one-out strategy for each dataset and optimize the model using the AdamW optimizer with a learning rate of $1\times 10^{-4}$. The batch size is set to 128, and training is performed for 300 k iterations. For preprocessing, each spot image is cropped to 224 × 224 pixels centered on the spot coordinates, which corresponds to a physical field of view of approximately 60–210 μm across the seven datasets used in this study, depending on the imaging resolution of each slide. This scale spans from a few cells to a few dozen cells around each spot, providing the model with the immediate cellular and micro-architectural context surrounding the profiled spot. Gene expression values are normalized by dividing by the total expression per spot and subsequently log-transformed. For parameter settings, the number of GNN layers is set to 2, the hidden dimension *d* is 384, and the scaling factor α is 0.5. The dimension of pretrained gene embeddings *E* is 200. In the sampling stage, we apply the Euler method as the ODE solver with 100 steps to generate the prediction. Details of training and sampling are reported in Appendix C. Following the evaluation protocol in (Xie *et al.* 2023), performance is assessed on the top 50 most Highly Variable Genes (HVGs) and the top 50 most Highly Expressed Genes (HEGs) using Pearson Correlation Coefficient (PCC) and Mean Squared Error (MSE).

### 3.2 Experimental results

#### 3.2.1 Quantitative comparison

Table 1 presents the quantitative performance of PSSD compared with baseline methods across three ST datasets. The evaluation focuses on the top 50 HVGs and HEGs, quantified using PCC. Overall, PSSD achieves the best performance on all datasets, demonstrating strong generalization capability. Specifically, on the DLPFC dataset, PSSD attains average PCC values of 0.337 for HVGs and 0.299 for HEGs, representing improvements of 12.3% and 11.9% over the diffusion-based method, Stem. On the BC dataset, PSSD also achieves superior PCCs of 0.369 for HVGs and 0.370 for HEGs. The advantage is more pronounced on the cSCC dataset, where PSSD achieves the highest PCC values of 0.416 (HVGs) and 0.411 (HEGs), significantly outperforming all baseline methods. To further assess generalization, Table 2 reports the PCC performance of PSSD and the four strongest baselines (ST-Net, BLEEP, Stem, STFlow) on three additional HEST-1k Visium datasets (ccRCC, PAAD, COAD), and Table 3 reports the corresponding evaluation on the higher-resolution Visium HD COAD dataset. Across both standard Visium and higher-resolution platforms, PSSD consistently maintains the leading PCC, confirming that the decoupled flow-based formulation transfers robustly to new tissues and resolutions. Comparisons with foundation models are provided in Fig. D.1 in Appendix D, available as supplementary data at *Bioinformatics* online. Quantitative comparisons of MSE, NRMSE, and SSIM are provided in Table D.5–Table D.13 and Fig. D.2 in Appendix D, available as supplementary data at *Bioinformatics* online.

**Figure 2:**
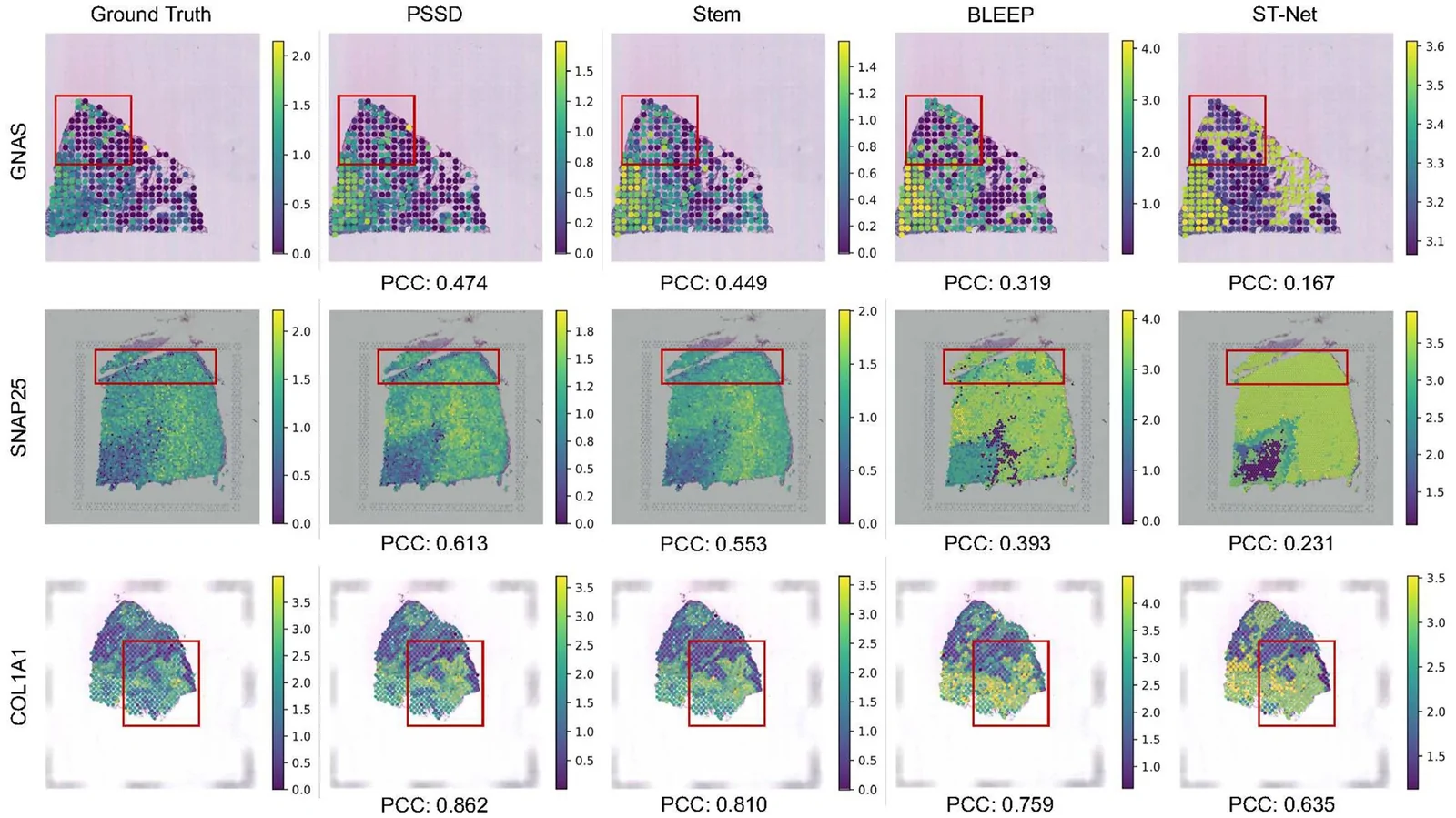
The qualitative comparison includes the ground truth and predicted biomarker gene expression levels generated by PSSD, Stem, BLEEP, and ST-Net across samples from BC, DLPFC, and cSCC datasets. Highlighted regions emphasize high spatial variance and irregular expression.

**Table 2:** Quantitative comparison of PSSD and baseline methods on three additional HEST-1k datasets.

Datasets	**ccRCC**		**PAAD**		**COAD**	
Methods	HVG	HEG	HVG	HEG	HVG	HEG
ST-Net (He *et al.* 2020)	0.217 ± 0.0988	0.290 ± 0.1353	0.168 ± 0.0781	0.388 ± 0.0625	0.316 ± 0.1335	0.394 ± 0.1546
BLEEP (Xie *et al.* 2023)	0.178 ± 0.0847	0.235 ± 0.1324	0.176 ± 0.0841	0.384 ± 0.0620	0.346 ± 0.1636	0.440 ± 0.1669
Stem (Zhu *et al.* 2025)	0.224 ± 0.0710	0.294 ± 0.1224	0.181 ± 0.0764	0.345 ± 0.0750	0.356 ± 0.1574	0.433 ± 0.1709
STFlow (Huang *et al.* 2025)	0.317 ± 0.0832	0.391 ± 0.1186	0.274 ± 0.1062	0.427 ± 0.0594	0.430 ± 0.1722	0.503 ± 0.1791
PSSD (Ours)	**0.334** ± **0.0540**	**0.416** ± **0.1056**	**0.291** ± **0.0915**	**0.454** ± **0.0524**	**0.458** ± **0.1542**	**0.535** ± **0.1623**

Reported values are Pearson Correlation Coefficients (PCC↑) for the top 50 Highly Variable Genes (HVGs) and top 50 Highly Expressed Genes (HEGs). The best results are in **bold**; the second-best results are underlined.

**Table 3:** Quantitative comparison of PSSD and baseline methods on the higher-resolution Visium HD COAD dataset.

Methods	HVG	HEG
ST-Net (He *et al.* 2020)	0.000 ± 0.0001	0.003 ± 0.0040
BLEEP (Xie *et al.* 2023)	0.002 ± 0.0147	0.005 ± 0.0041
Stem (Zhu *et al.* 2025)	−0.003 ± 0.0107	0.005 ± 0.0028
STFlow (Huang *et al.* 2025)	0.029 ± 0.0086	0.034 ± 0.0131
PSSD (Ours)	**0.037** ± **0.0072**	**0.042** ± **0.0118**

Reported values are PCC↑ for the top 50 HVGs and top 50 HEGs. The best results are in **bold**; the second-best results are underlined.

#### 3.2.2 Qualitative comparison

To ensure the reliability for downstream morphological profiling, we validate the biological consistency of PSSD. Several representative biomarker genes (He *et al.* 2020, Forsthuber *et al.* 2024, Lei *et al.* 2025, Wei *et al.* 2025), including GNAS, IGHA1, SNAP25, MBP, COL1A1, and KRT6A, are selected to assess whether the model preserves functional tissue microenvironments. Figure 2 visualizes GNAS, SNAP25, and COL1A1 (with additional examples in Appendix D, Fig. D.3, available as supplementary data at *Bioinformatics* online). For GNAS, a crucial marker at highly heterogeneous tissue interfaces, PSSD reconstructs the sharp biological boundaries more faithfully than the compared baselines. In contrast, baselines (e.g. BLEEP, ST-Net) tend to over-smooth these regions and fail to preserve the finer regional structure, which, as we quantify in the downstream tissue domain identification experiment in Appendix D (Table D.19 and Fig. D.4, available as supplementary data at *Bioinformatics* online), translates into noticeably lower agreement with the SpatialLIBD cortical layer annotations. For SNAP25, a neuronal marker in DLPFC, PSSD visually captures the fine-grained heterogeneity of the neuronal domain more closely than the two strongest generative baselines. This qualitative behaviour is supported at the population level by the marker-gene spatial autocorrelation analysis in Appendix D (Table D.20 and Fig. D.5, available as supplementary data at *Bioinformatics* online), where PSSD’s Moran’s I is systematically the closest to the ground truth across seven representative DLPFC cortical layer markers, indicating that our decoupled semantic flow better preserves genes with heterogeneous spatial distributions rather than only fitting a small number of hand-picked examples. Moreover, for the stroma-enriched COL1A1, PSSD tends to preserve a more localized stromal signal than the compared baselines, while we acknowledge that the visual differences between methods on this gene are more subtle than on GNAS or SNAP25, and are best interpreted alongside the quantitative per-panel PCC now reported in Figs 2 and D.3, available as supplementary data at *Bioinformatics* online. These observations demonstrate that PSSD provides biologically meaningful and structurally coherent representations driven by the dynamic trajectory optimization of our progressive decoupling network. To further quantify how well the predicted expression supports biologically meaningful downstream analyses, a tissue domain identification experiment on the SpatialLIBD cortical layer annotations of the DLPFC dataset is provided in Appendix D (Table D.19 and Fig. D.4, available as supplementary data at *Bioinformatics* online), together with a marker-gene spatial autocorrelation analysis (Table 500.20 and Figure 500.5, available as supplementary data at *Bioinformatics* online), where PSSD substantially outperforms the two strongest generative baselines.

**Figure 3:**
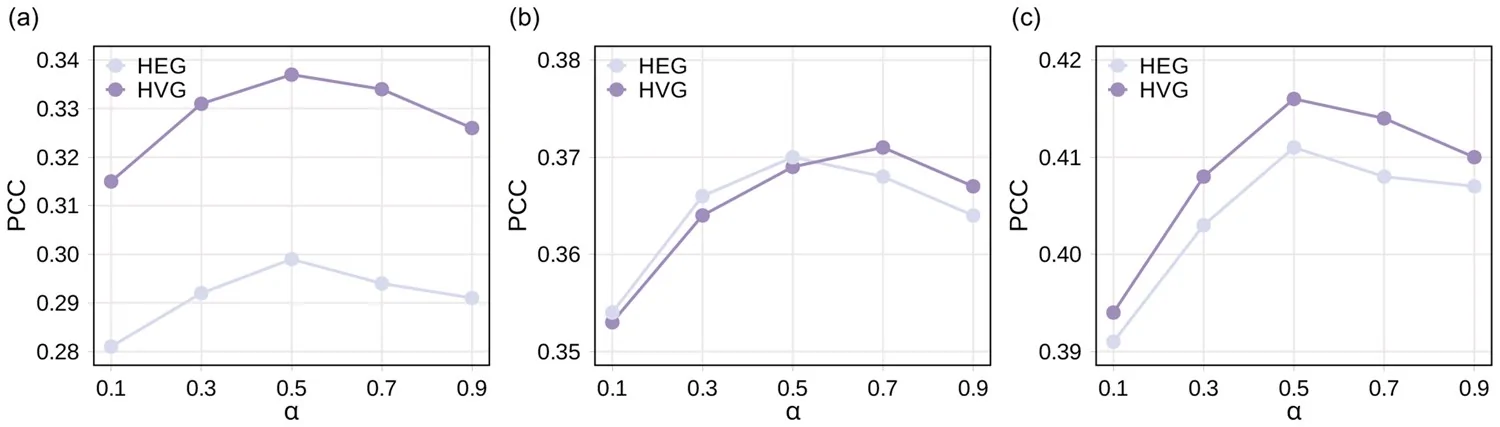
Performance of PSSD with varying values of hyperparameter α on (a) DLPFC, (b) BC, and (c) cSCC datasets by the measurement of PCC.

**Figure 4:**
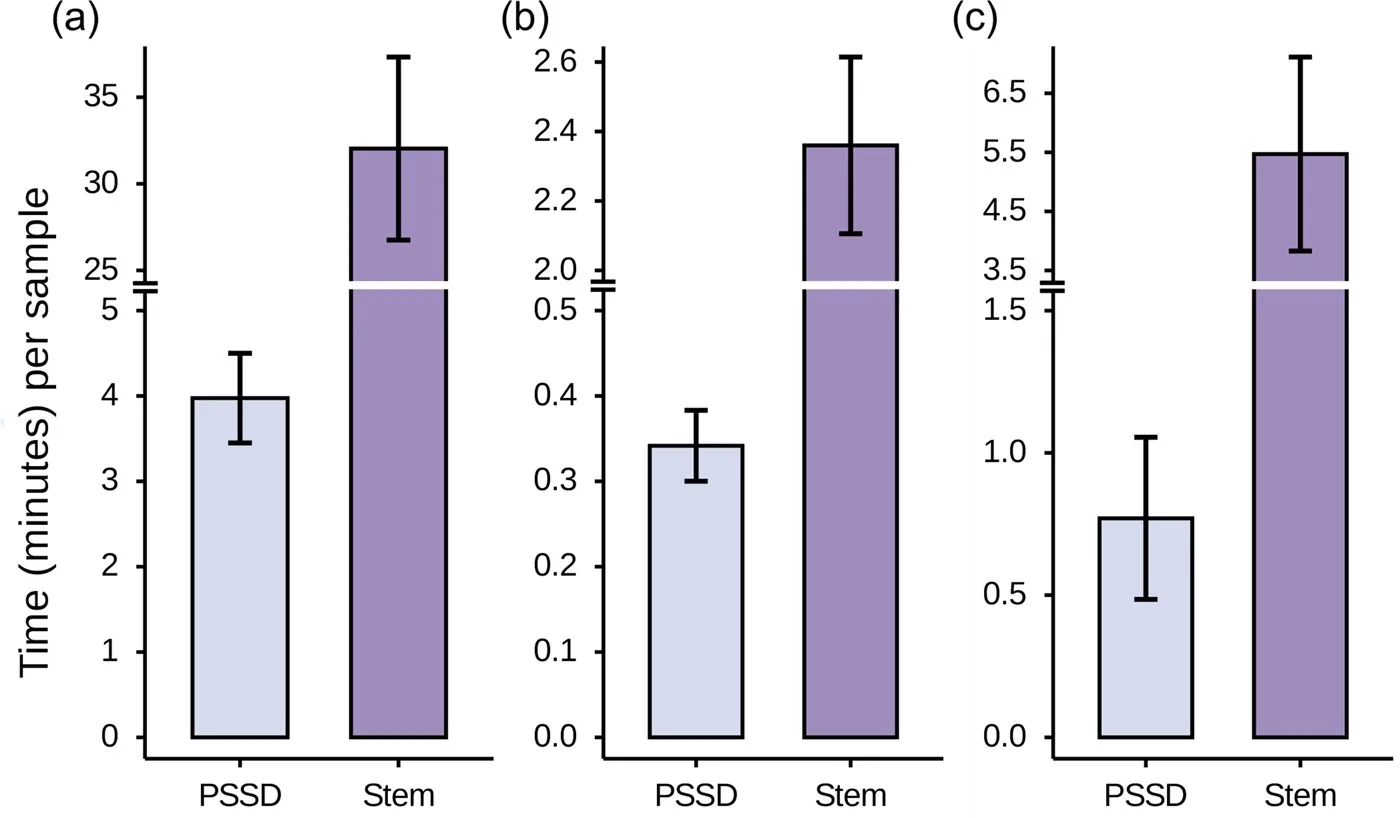
The time required for a single-sample inference per slide for PSSD and Stem on the (a) DLPFC, (b) BC, and (c) cSCC datasets.

#### 3.2.3 Ablation studies

Table 4 reports the ablation results on three datasets to assess the contribution of both the generative paradigm and key architectural components. Crucially, substituting the flow-based ODE engine with a diffusion process (PSSD-Diffusion) leads to a marked performance drop. This confirms that the deterministic, linear trajectory of flow matching is mathematically required for our framework; the high-variance noise intrinsic to standard diffusion severely destabilizes our gating mechanism. Architecturally, removing the decoupling structure leads to the most significant performance degradation, proving the absolute necessity of disentangling spatial and semantic representations for accurate gene prediction. Furthermore, the removal of the biological prior and adaptive fusion results in consistent drops, indicating that static biological anchors and dynamic trajectory integration are essential for capturing spatially coherent expression. Finally, excluding the gating mechanism weakens performance, demonstrating that controlled cross-stream interaction is critical for stabilizing representation learning. These results collectively verify that the continuous flow matching and the decoupling architecture are inextricably linked to achieve PSSD’s superior performance.

**Table 4:** Ablation studies on the generative paradigm and key architectural components in PSSD, measured by average PCC for the top 50 HVGs and HEGs.

Datasets	DLPFC		BC		cSCC	
Model Variant	HVG	HEG	HVG	HEG	HVG	HEG
*Ablation on Architecture*						
w/o Biological Prior	0.312	0.287	0.352	0.360	0.394	0.388
w/o Decoupling	0.309	0.281	0.350	0.353	0.379	0.373
w/o Gating	0.321	0.296	0.362	0.364	0.401	0.393
w/o Adaptive Fusion	0.326	0.294	0.364	0.366	0.404	0.401
*Ablation on Generative Paradigm*						
PSSD (DDPM)	0.320	0.285	0.358	0.361	0.395	0.390
**PSSD (Flow Matching)**	**0.337**	**0.299**	**0.369**	**0.370**	**0.416**	**0.411**

**Table 5:** Performance on Decoupled Features vs. Moran’s I.

Representative Gene	Moran’s I	PCCsp	PCCse	Gap
MBP	0.518	0.437	0.386	+0.051
SNAP25	0.311	0.425	0.389	+0.036
FASN	0.011	0.328	0.340	−0.012
CD74	0.100	0.307	0.322	−0.015

#### 3.2.4 Effect of hyperparameter

α Fig. 3 illustrates the effect of varying the hyperparameter α on model performance across DLPFC, BC, and cSCC datasets. α controls the balance between spatial and semantic information during joint optimization. As α increases from 0.1 to 0.5, the average PCC improves steadily, reaching the highest values around α=0.5 for both HVG and HEG across all datasets. This indicates that a moderate weighting effectively harmonizes the two learning objectives, enabling the model to capture both local spatial continuity and global gene-level semantics. When α further increases beyond 0.7, performance slightly declines. Nevertheless, PSSD maintains stable performance across a wide range of α values, demonstrating its robustness to hyperparameter variation.

#### 3.2.5 Empirical analysis of decoupling

To validate that our decoupling architecture captures biological mechanisms rather than computational artifacts, Table 5 presents a gene-specific linear probe analysis. We measure the predictive performance gap (PCCsp—PCCse) between the isolated spatial and semantic flows and correlate it with the intrinsic spatial autocorrelation (Moran’s I) of representative genes. Crucially, the spatial flow dominates for genes exhibiting high Moran’s I (e.g. MBP, +0.051 gap), confirming its role in preserving morphological continuity. Conversely, the semantic flow excels at reconstructing heterogeneous genes with low spatial dependence (e.g. CD74, −0.015 gap). This biological validation proves that our progressive decoupling mechanism does not arbitrarily separate latent features, but disentangles the underlying biological drivers of spatial continuity and functional heterogeneity.

#### 3.2.6 Comparison of generation time

In Fig. 4, we compare the generation efficiency of PSSD against the diffusion-based method Stem. On the DLPFC, BC, and cSCC datasets, PSSD requires only 3.98, 0.34, and 0.77 minutes per sample on average, whereas Stem requires 32.04, 2.36, and 5.47 min, respectively. This nearly eightfold acceleration results from the flow matching mechanism, which directly models deterministic transport rather than relying on iterative diffusion steps. These results demonstrate that PSSD not only achieves high predictive accuracy but also offers superior scalability for large-scale spatial transcriptomics generation. A full training-plus-inference cost comparison against all four baselines on a single NVIDIA RTX 4090 GPU, together with each method’s default sampling protocol (integration steps and number of samples averaged per spot), is reported in Table E.22 of Appendix E, available as supplementary data at *Bioinformatics* online. Under the shared 100-step 20-sample protocol used to produce the PCC/ARI/Moran’s-I results in this paper, PSSD retains a substantial sampling-speed advantage over Stem while achieving markedly better PCC (Table 1), ARI (Table D.19, available as supplementary data at *Bioinformatics* online), and Moran’s-I fidelity (Table D.20, available as supplementary data at *Bioinformatics* online).

## 4 Conclusion

This work presents PSSD, a novel generative framework that leverages conditional flow matching and Progressive Spatial-Semantic Decoupling to predict spatial gene expression from histology images. Moving beyond static feature-level compromise, PSSD formulates cross-modal integration as a dynamic trajectory optimization problem, successfully resolving the inherent tension between spatial continuity and functional heterogeneity. Consequently, the model not only achieves state-of-the-art predictive accuracy and an 8x sampling acceleration, but also preserves sharp, biologically faithful tissue boundaries critical for downstream morphological profiling. A promising future direction lies in extending PSSD to single-cell–resolution ST, where efficiently optimizing the decoupling strategy under finer spatial scales remains a challenging problem for more comprehensive spatial omics analysis.

## Supplementary Material

btag598_Supplementary_Data

## Data Availability

The code and data underlying this article are available in https://github.com/ChyaZhang/PSSD.
