# Supplementary material for: PSSD: Progressive Spatial-Semantic Decoupling for flow-based gene expression prediction from histology images
Source: Bioinformatics. 2026 Aug 8;42(8):btag598. doi: 10.1093/bioinformatics/btag598 (PMC13506047; doi:10.1093/bioinformatics/btag598)
Supplement: Supplementary file 1 [file btag598_supplementary_data.pdf]

Table A.1. Overview of ID, spot count, and gene count in the DLPFC, BC, and cSCC Datasets.

DLPFC			BC			cSCC		
ID in HEST-1k	Spot Count	Gene Count	ID in HEST-1k	Spot Count	Gene Count	ID in HEST-1k	Spot Count	Gene Count
MISC1	3460	33538	SPA143	277	15387	NCBI759	462	17047
MISC2	3592	33538	SPA144	289	15273	NCBI760	621	16642
MISC3	3673	33538	SPA145	283	15289	NCBI761	608	15383
MISC4	3639	33538	SPA146	298	15215	NCBI762	1182	17976
MISC5	4015	33538	SPA147	270	15290	NCBI763	1071	19314
MISC6	4110	33538	SPA148	295	15109	NCBI764	1145	17823
MISC7	3498	33538	SPA149	360	15645	NCBI765	521	17399
MISC8	3661	33538	SPA150	332	15638	NCBI766	521	17689
MISC9	4634	33538	SPA151	343	15538	NCBI767	590	16959
MISC10	4789	33538	SPA152	359	15517	NCBI768	638	17883
MISC11	4384	33538	SPA153	325	15526	NCBI769	646	17344
MISC12	4226	33538	SPA154	346	15045	NCBI770	666	17138

Table A.2. Overview of ID, spot count, and gene count for the three additional HEST-1k datasets used to assess the generalization of PSSD. PAAD contains only four publicly available slices; the remaining rows are marked as "–".

ccRCC			PAAD			COAD		
ID in HEST-1k	Spot Count	Gene Count	ID in HEST-1k	Spot Count	Gene Count	ID in HEST-1k	Spot Count	Gene Count
INT13	4359	17943	NCBI569	3493	20615	MISC62	4132	19391
INT14	4562	17943	NCBI570	3216	20615	MISC63	3467	19346
INT15	4940	17943	NCBI571	2498	20615	MISC64	2533	19411
INT16	3206	17943	NCBI572	3794	20615	MISC65	3261	19402
INT17	3585	17943	–	–	–	MISC66	3970	19347
INT18	4915	17943	–	–	–	MISC67	2551	19384
INT19	4948	17943	–	–	–	MISC68	2708	19359
INT20	4860	17943	–	–	–	MISC69	3634	19263
INT21	4975	17943	–	–	–	MISC70	3934	19116
INT22	3829	17943	–	–	–	MISC71	2997	19251
INT23	4755	17943	–	–	–	MISC72	4369	19310
INT24	4510	17943	–	–	–	MISC73	3901	19074

Table A.3. Overview of ID, spot count, and gene count for the higher-resolution Visium HD COAD dataset.

Visium HD COAD		
ID in HEST-1k	Spot Count	Gene Count
TENX154	2220	18085
TENX155	2281	18085
TENX156	2179	18085

Table A.4. Dataset composition: patient and sample counts for all evaluated cohorts.

Dataset	Samples	Patients
DLPFC	12	1
BC	12	2
cSCC	12	4
ccRCC	12	12
PAAD	4	—
COAD	12	1
Visium HD COAD	3	3

A. Details of Compared Methods and Datasets

To demonstrate the effectiveness of PSSD, we compare it with nine state-of-the-art spatial gene expression prediction methods and four pathology foundation models on three ST datasets. The ID, spot count, and gene count of each slice is listed in Table A.1. The corresponding statistics for the three additional HEST-1k datasets (ccRCC, PAAD, COAD) used to evaluate cross-tissue generalization are reported in Table A.2, and those for the higher-resolution Visium HD COAD dataset are reported in Table A.3. Table A.4 declares the dataset composition in the experiments. Detailed descriptions of the compared methods are summarized below:

Hist2ST (Zeng et al., 2022): A deep learning framework that integrates convolutional, Transformer, and graph neural network modules to capture both visual and spatial dependencies among tissue spots.

HisToGene (Pang et al., 2021): A deep learning method that predicts spatial gene expression directly from histology images by leveraging a Vision Transformer to capture spatial dependencies among tissue spots.

IHItoGene (Jia et al., 2023): A hybrid neural network employing dynamic convolutional and capsule networks to extract molecular signals from histological images and model the relationship between tissue phenotypes and gene regulation.

ST-Net (He et al., 2020): A method that accurately identifies key biomarkers and generalizes across external datasets, enabling image-based molecular profiling without retraining.

TRIPLEX (Chung et al., 2024): A gene prediction method that integrates multi-resolution features, including cellular, local, and global tissue contexts.

TANGLE (Jaume et al., 2024b): A multi-modal pretraining framework that learns whole-slide image representations by

aligning histopathology and gene expression modalities through contrastive learning.

BLEEP (Xie et al., 2023): A bi-modal embedding method that constructs a low-dimensional joint embedding space using paired image and expression profiles.

M2TGLGO (Shi et al., 2025): A multi-modal method that predicts spatially resolved gene expression from pathology images by integrating deep and handcrafted features while preserving the spatial topology of tissue spots.

Stem (Zhu et al., 2025): A conditional diffusion model that captures the stochasticity and heterogeneity of spatial transcriptomics data to predict gene expression from histology images.

CONCH (Lu et al., 2024): A visual-language foundation model pretrained on over 1.17 million histopathology image–caption pairs for task-agnostic learning, integrating both image and textual information.

UNI (Chen et al., 2024): A self-supervised method pretrained on more than 100 million H&E-stained whole-slide images across 20 tissue types to learn general-purpose representations for computational pathology.

GigaPath (Xu et al., 2024): A vision transformer framework pretrained on gigapixel whole-slide pathology images, efficiently modeling tens of thousands of tiles per slide using the LongNet approach.

Virchow2 (Zimmermann et al., 2024): A vision transformer model with 632 million parameters pretrained on large-scale, diverse histopathology datasets, achieving state-of-the-art performance across multiple computational pathology tasks.

To ensure a fair evaluation, our experimental setup follows the benchmark protocol established by the recent state-of-the-art method M2TGLGO (Shi et al., 2025). We trained baseline methods using their official implementations and default optimal hyperparameter settings as validated in their original literature.

B. Neural Network Architecture

Our neural network parameterizes the vector field in the flow matching and is constructed upon DiT blocks (adaLN-Zero design) and decoupled blocks. Across all of our experiments, the network contains 12 blocks in total: the first four and last four are DiT blocks, while the middle four are decoupled blocks. Each block includes 6 attention heads and a hidden dimension of 384. The output of the final DiT block is fed to a lightweight decoder consisting of an adaLN layer and a linear layer that produces a one-dimensional output. SiLU is used as the activation function for all MLPs within the network.

C. Training and Inference Dynamics

Training Dynamics During training, the state variable $x_t = (1-t)x_0 + tx_1$ is a linear interpolation between pure Gaussian noise x_0 and the ground-truth gene expression x_1. The visual embeddings z and spatial coordinates s act as morphological conditions. Importantly, the biological prior h^{corr} (derived from Gene2Vec and the GO graph) serves as a static semantic anchor. Even in high-noise regimes (e.g., $t \to 0$), h^{corr} provides deterministic relational guidance, compelling the predicted vector field v_θ to respect established gene-gene interaction topologies rather than overfitting to noise correlations.

Inference Dynamics During inference, the ground-truth expression x_1 is inaccessible, and generation initializes from pure noise $x_0 \sim \mathcal{N}(0, I)$. At this stage, the ODE trajectory is entirely steered by the cross-stream interaction between the visual-spatial condition (z, s) and the static semantic anchor (h^{corr}). The adaptive fusion gate evaluates the local spatial context at each integration step t, deciding whether the generative trajectory should be governed by the spatial flow or the semantic flow. This dual-path guidance ensures strict biological fidelity without relying on any ground-truth leakage.

D. Additional Experiments

Quantitative comparison of MSE Table D.5 summarizes the quantitative comparison between PSSD and existing approaches on the DLPFC, BC, and cSCC datasets, evaluated using MSE for both HVGs and HEGs. Although PSSD and Stem are generative models that are not directly optimized for MSE, PSSD still achieves the lowest or second-lowest MSE in some cases. In particular, PSSD attains notable improvements on the DLPFC dataset, achieving 0.078 for HVGs. While deterministic regression methods such as M2TGLGO slightly outperform PSSD due to their loss-specific optimization, the overall MSE gap remains small. More importantly, PSSD demonstrates a substantial advantage in PCC (Table 1), suggesting that it generates biologically coherent and spatially consistent expression profiles rather than merely minimizing numerical reconstruction error. Tables D.6 and D.7 extend the MSE comparison to the three additional HEST-1k datasets (ccRCC, PAAD, COAD) and the higher-resolution Visium HD COAD dataset, where PSSD remains highly competitive against the strongest baselines (ST-Net, BLEEP, Stem, STFlow).

Quantitative comparison of NRMSE and SSIM To provide a more complete assessment beyond linear correlation and raw squared error, we further evaluate range-normalized RMSE (NRMSE) and Structural Similarity Index (SSIM), which together capture relative reconstruction error and the preservation of local spatial structure, respectively. Tables D.8 and D.11 report NRMSE and SSIM on the DLPFC, BC, and cSCC datasets; Tables D.9 and D.12 report the same metrics on the three additional HEST-1k datasets (ccRCC, PAAD, COAD); and Tables D.10 and D.13 report them on the higher-resolution Visium HD COAD dataset. Across all metrics, datasets, and platforms, PSSD consistently achieves the most favorable balance, indicating that its decoupled flow formulation does not merely improve PCC but also preserves absolute expression magnitude and local spatial coherence.

Extension to progressively larger gene sets To verify that the advantage of PSSD is not restricted to the top 50 gene subset used for consistency with prior work, we extend the evaluation to top 100 and top 200 HVGs and HEGs on the DLPFC, BC, and cSCC datasets. The comparison uses the two strongest generative baselines (Stem and STFlow) whose training pipeline covers the same top-200 gene pool, allowing a like-for-like evaluation. As shown in Table D.14, PSSD consistently attains the highest PCC at every gene-set size on both HVGs and HEGs. The PCC of every method decreases as the gene set grows, which is expected because the additional genes have

Table D.5. Quantitative comparison of PSSD and baseline methods on three ST datasets. Reported values are Mean Squared Error (MSE↓) for the top 50 Highly Variable Genes (HVGs) and top 50 Highly Expressed Genes (HEGs). The best results are in **bold**; the second-best results are <u>underlined</u>.

Datasets	DLPFC		BC		cSCC	
Methods	HVG	HEG	HVG	HEG	HVG	HEG
Hist2ST (Zeng et al., 2022)	0.234 ± 0.0299	0.296 ± 0.0053	0.271 ± 0.0873	0.237 ± 0.0136	0.324 ± 0.1284	0.312 ± 0.1103
HisToGene (Pang et al., 2021)	0.207 ± 0.0701	0.249 ± 0.1182	0.353 ± 0.1095	0.313 ± 0.0012	0.291 ± 0.0520	0.242 ± 0.1405
IHItoGene (Jia et al., 2023)	0.341 ± 0.0123	0.311 ± 0.0499	0.301 ± 0.0694	0.299 ± 0.0071	0.287 ± 0.0795	0.265 ± 0.0043
ST-Net (He et al., 2020)	0.292 ± 0.1344	0.307 ± 0.0341	0.258 ± 0.0128	0.251 ± 0.0108	0.293 ± 0.0385	0.232 ± 0.0154
TRIPLEX (Chung et al., 2024)	0.225 ± 0.0556	0.219 ± 0.0536	0.226 ± 0.0959	0.212 ± 0.1235	0.258 ± 0.0875	0.202 ± 0.0751
TANGLE (Jaume et al., 2024b)	0.214 ± 0.0307	0.209 ± 0.0416	0.262 ± 0.0131	0.284 ± 0.0762	0.227 ± 0.0265	0.259 ± 0.0394
BLEEP (Xie et al., 2023)	0.181 ± 0.0759	0.134 ± 0.0679	0.206 ± 0.0430	<u>0.168 ± 0.0343</u>	0.238 ± 0.0788	0.214 ± 0.1003
M2TGLGO (Shi et al., 2025)	0.130 ± 0.0185	**0.101 ± 0.0217**	**0.157 ± 0.0313**	**0.137 ± 0.0172**	<u>0.184 ± 0.0274</u>	**0.141 ± 0.0284**
Stem (Zhu et al., 2025)	<u>0.094 ± 0.0093</u>	0.137 ± 0.0190	0.191 ± 0.1035	0.197 ± 0.1068	0.195 ± 0.0812	0.270 ± 0.1275
STFlow (Huang et al., 2025)	0.103 ± 0.0132	0.131 ± 0.0149	0.196 ± 0.0982	0.205 ± 0.1127	0.187 ± 0.0913	0.233 ± 0.1143
PSSD (Ours)	**0.078 ± 0.0125**	<u>0.126 ± 0.0158</u>	<u>0.188 ± 0.1021</u>	0.195 ± 0.1057	**0.179 ± 0.0746**	<u>0.184 ± 0.0466</u>

Table D.6. Quantitative comparison of PSSD and baseline methods on three additional HEST-1k datasets. Reported values are Mean Squared Error (MSE↓) for the top 50 HVGs and top 50 HEGs. The best results are in **bold**; the second-best results are <u>underlined</u>.

Datasets	ccRCC		PAAD		COAD	
Methods	HVG	HEG	HVG	HEG	HVG	HEG
ST-Net (He et al., 2020)	**3.131 ± 0.9568**	<u>2.135 ± 0.4559</u>	<u>5.238 ± 2.3703</u>	<u>1.812 ± 0.2320</u>	<u>4.721 ± 2.9264</u>	<u>4.372 ± 3.0998</u>
BLEEP (Xie et al., 2023)	3.749 ± 0.9629	2.498 ± 0.2900	5.291 ± 2.2606	1.827 ± 0.2424	4.911 ± 2.4976	4.573 ± 3.6744
Stem (Zhu et al., 2025)	3.281 ± 0.9402	2.214 ± 0.4289	5.914 ± 2.4689	1.889 ± 0.2712	**4.699 ± 2.8653**	4.378 ± 3.2401
STFlow (Huang et al., 2025)	3.360 ± 0.9480	2.562 ± 0.5520	5.785 ± 2.0250	2.022 ± 0.2790	4.786 ± 2.8620	4.422 ± 3.5870
PSSD (Ours)	<u>3.162 ± 0.9320</u>	**2.078 ± 0.4400**	**5.080 ± 2.3100**	**1.755 ± 0.2205**	4.733 ± 2.7800	**4.228 ± 3.0100**

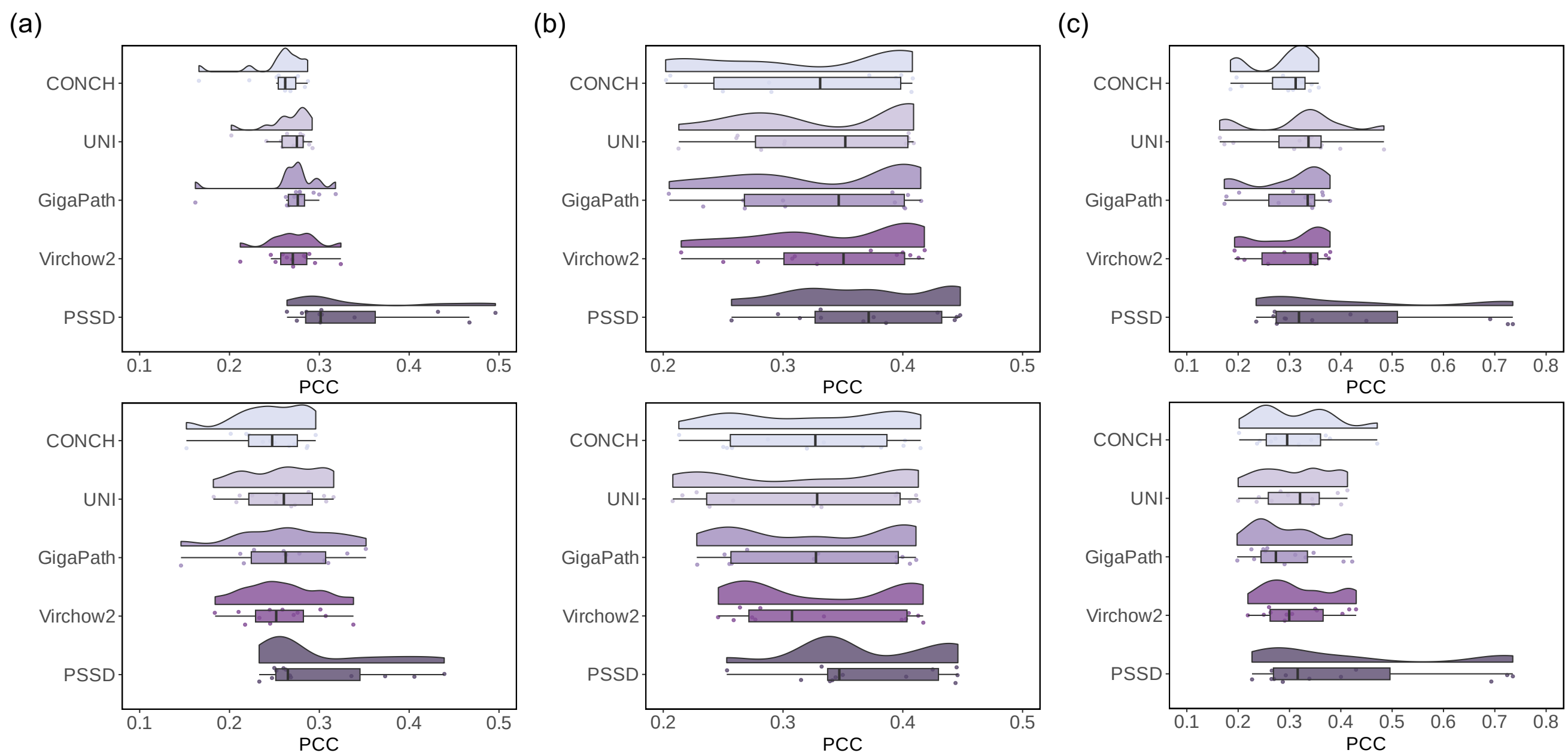

Figure D.1 Quantitative comparison of PSSD with four pathology foundation models on (a) DLPFC, (b) BC, and (c) cSCC datasets by the measurement of average PCC for the top 50 HVGs (first row) and top 50 HEGs (second row).

lower signal-to-noise ratio and are harder to predict. Importantly, this decrease is markedly smaller for PSSD (approximately 12% from top 50 to top 200 on DLPFC HVG) than for STFlow (approximately 20%) and Stem (approximately 24%). As a result, the relative advantage of PSSD does not shrink and even slightly widens as more genes are considered, indicating that the progressive spatial–semantic decoupling learns a globally coherent representation rather than one that is only well-tuned for the strongest few genes.

Quantitative comparison with foundation models Figure D.1 visualizes the PCC distributions of PSSD and four pathological foundation models. Across all three datasets, the mean PCC of PSSD consistently exceeds that of the four foundation models. However, regarding median and variance, PSSD underperforms some models in specific datasets, indicating

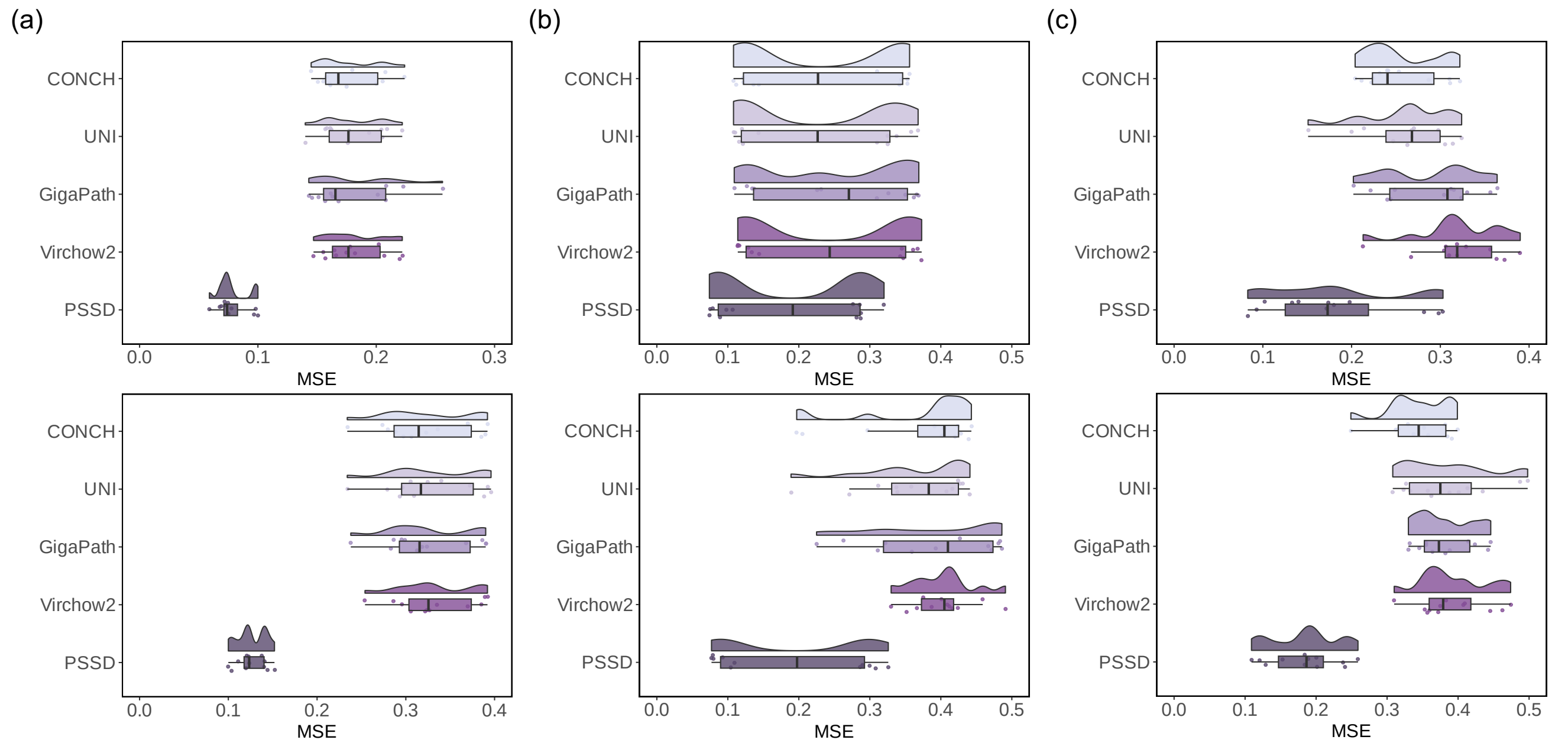

Figure D.2 Quantitative comparison of PSSD with four pathology foundation models on (a) DLPFC, (b) BC, and (c) cSCC datasets by the measurement of average MSE for the top 50 HVGs (first row) and top 50 HEGs (second row).

Table D.7. Quantitative comparison of PSSD and baseline methods on the Visium HD COAD dataset. Reported values are MSE↓ for the top 50 HVGs and top 50 HEGs.

Methods	HVG	HEG
ST-Net (He et al., 2020)	**1.598 ± 0.3272**	2.234 ± 0.4196
BLEEP (Xie et al., 2023)	1.815 ± 0.3059	2.489 ± 0.5086
Stem (Zhu et al., 2025)	1.635 ± 0.2861	2.204 ± 0.2407
STFlow (Huang et al., 2025)	1.770 ± 0.4870	2.722 ± 0.8110
PSSD (Ours)	1.612 ± 0.3160	**2.195 ± 0.2335**

potential improvement in predictive stability. Figure D.2 further visualizes the MSE distributions of PSSD and four pathological foundation models. Across three datasets, the mean MSE of PSSD consistently remains lower than that of the foundation models. These results indicate that PSSD achieves a superior balance between spatial fidelity and expression accuracy, underscoring the effectiveness of its flow-based decoupling framework.

Impact of Image Encoder In this ablation study, we evaluate the effect of different pathological foundation models used as image feature encoders on PSSD performance. The considered models included CONCH (Lu et al., 2024), UNI (Chen et al., 2024), and GigaPath (Xu et al., 2024). These three models produce embeddings with dimensions of 512, 1,024, and 1,536, respectively, from 224 × 224 image patches. Virchow2 (Zimmermann et al., 2024) is excluded because its 2,048-dimensional embeddings substantially increase training cost. We evaluate the following settings on the BC dataset (Andersson et al., 2020), including the single use of each model and the combination of CONCH and UNI, as summarized in Table D.15. The results indicate that combining embeddings from CONCH and UNI as visual features yields the best PSSD performance. It is worth noting that the combined embedding dimension of CONCH and UNI matches that of GigaPath. However, given the distinct training data of the base models, the combined embedding is expected to contain richer information, thereby achieving superior performance. Under the consideration of training efficiency, we do not further examine the effects of combining embeddings from additional foundation models, and this will be explored in future work.

Choice of ODE solver As shown in Table D.16, different ODE solvers exhibit varying trade-offs between generation accuracy and efficiency. The Euler solver achieves the fastest generation speed across all datasets, requiring only 3.98, 0.34, and 0.77 minutes per sample on DLPFC, BC, and cSCC, respectively, while maintaining competitive PCC performance comparable to more complex solvers. In contrast, higher-order methods such as RK4 and Midpoint preserve accuracy but incur a $2-4\times$ increase in computational cost. Heun achieves the best PCC on cSCC (0.417) but remains slower than Euler. Considering both accuracy and efficiency, the Euler solver provides the optimal balance, confirming that the proposed PSSD framework remains robust and effective under a simple numerical integration scheme.

Impact of gene neighbors Table D.17 presents the ablation results on the number of gene neighbors. As the number increases from 4 to 10, the average PCC consistently improves across all datasets. Specifically, using 10 neighbors yields the highest PCC in most settings. However, further increasing the number to 12 results in marginal or no improvement, suggesting that excessive neighborhood aggregation may introduce noise. Overall, a setting of 10 neighbors provides the best trade-off between capturing gene co-expression structure and preserving local specificity.

Effect of the learnable gate λ To prevent spatial bias, our adaptive fusion dynamically regulates spatial dependence via a learnable gate λ. We validate this via coordinate perturbation test on the DLPFC dataset, as shown in Table D.18. Crucially,

Table D.8. Quantitative comparison of PSSD and baseline methods on the DLPFC, BC, and cSCC datasets. Reported values are range-normalized Root Mean Squared Error (NRMSE↓) for the top 50 HVGs and top 50 HEGs. The best results are in **bold**; the second-best results are underlined.

Datasets	DLPFC		BC		cSCC	
Methods	HVG	HEG	HVG	HEG	HVG	HEG
ST-Net (He et al., 2020)	0.165 ± 0.0148	0.192 ± 0.0223	0.175 ± 0.0187	0.178 ± 0.0194	0.235 ± 0.0158	0.258 ± 0.0201
BLEEP (Xie et al., 2023)	0.158 ± 0.0142	0.180 ± 0.0206	0.170 ± 0.0173	0.172 ± 0.0181	0.225 ± 0.0146	0.248 ± 0.0189
Stem (Zhu et al., 2025)	0.150 ± 0.0133	0.168 ± 0.0195	0.162 ± 0.0164	0.164 ± 0.0170	0.212 ± 0.0134	0.235 ± 0.0180
STFlow (Huang et al., 2025)	0.148 ± 0.0131	**0.163 ± 0.0188**	0.159 ± 0.0160	0.160 ± 0.0165	0.208 ± 0.0130	**0.230 ± 0.0175**
PSSD (Ours)	**0.142 ± 0.0125**	0.166 ± 0.0180	**0.153 ± 0.0152**	**0.155 ± 0.0158**	**0.198 ± 0.0122**	0.237 ± 0.0166

Table D.9. Quantitative comparison of PSSD and baseline methods on three additional HEST-1k datasets. Reported values are NRMSE↓ for the top 50 HVGs and top 50 HEGs. The best results are in **bold**; the second-best results are underlined.

Datasets	ccRCC		PAAD		COAD	
Methods	HVG	HEG	HVG	HEG	HVG	HEG
ST-Net (He et al., 2020)	0.308 ± 0.0625	**0.213 ± 0.0236**	0.312 ± 0.0777	0.150 ± 0.0113	0.321 ± 0.1968	0.270 ± 0.1649
BLEEP (Xie et al., 2023)	0.349 ± 0.0729	0.232 ± 0.0259	0.322 ± 0.0956	0.151 ± 0.0144	**0.303 ± 0.1767**	0.271 ± 0.1672
Stem (Zhu et al., 2025)	0.317 ± 0.0576	0.220 ± 0.0263	0.323 ± 0.0619	0.154 ± 0.0144	0.310 ± 0.1820	0.264 ± 0.1612
STFlow (Huang et al., 2025)	0.295 ± 0.0598	0.269 ± 0.0452	0.301 ± 0.0459	0.159 ± 0.0151	0.356 ± 0.1026	**0.260 ± 0.1890**
PSSD (Ours)	**0.273 ± 0.0510**	0.216 ± 0.0210	**0.279 ± 0.0398**	**0.145 ± 0.0105**	0.307 ± 0.1650	0.262 ± 0.1520

Table D.10. Quantitative comparison of PSSD and baseline methods on the Visium HD COAD dataset. Reported values are NRMSE↓ for the top 50 HVGs and top 50 HEGs.

Methods	HVG	HEG
ST-Net (He et al., 2020)	**0.235 ± 0.0058**	0.256 ± 0.0055
BLEEP (Xie et al., 2023)	0.252 ± 0.0124	0.274 ± 0.0208
Stem (Zhu et al., 2025)	0.239 ± 0.0037	0.261 ± 0.0067
STFlow (Huang et al., 2025)	0.250 ± 0.0201	0.287 ± 0.0224
PSSD (Ours)	0.236 ± 0.0052	**0.248 ± 0.0050**

we observe that under such perturbations, the fusion coefficient λ automatically shifts to prioritize the semantic flow $\bar{h}^{se}$ (lowering spatial weight), confirming the model effectively suppresses unreliable spatial priors rather than overfitting to them.

Additional visualizations Figure D.3 presents additional qualitative comparisons of representative marker genes across multiple datasets, including GNAS, IGHA1, SNAP25, MBP, COL1A1, and KRT6A. Consistent with the results shown in Figure 2, PSSD demonstrates superior spatial fidelity and boundary preservation, effectively maintaining biologically meaningful spatial heterogeneity compared with existing baseline methods.

Downstream tissue domain identification To assess whether the predicted expression preserves the biological signal required for common downstream analyses, we perform a tissue domain identification experiment on the DLPFC dataset, which provides manually curated cortical layer annotations from the SpatialLIBD project (Maynard et al., 2021). For each of the 12 leave-one-out held-out slides, we cluster the predicted top 50 HVG expression using K-means with $K = 7$ (six cortical layers plus white matter) after PCA reduction, and match the resulting clusters to the reference annotations via the Hungarian assignment. Clustering quality is measured by Adjusted Rand Index (ARI) and Normalised Mutual Information (NMI). As reported in Table D.19, PSSD substantially outperforms both generative baselines, achieving 32% higher ARI and 20% higher NMI than Stem. Figure D.4 illustrates the spatial clustering on two representative slides. Consistent with the quantitative table, STFlow performs worse than Stem on this task despite its higher PCC on gene expression, which we attribute to STFlow's local spatial attention over-smoothing the predicted expression and blurring the cortical layer boundaries. In contrast, the progressive spatial-semantic decoupling in PSSD preserves both spot-wise expression fidelity and layer discriminability, confirming that the decoupled formulation directly benefits biologically meaningful downstream analyses.

Marker gene spatial autocorrelation To further examine whether the predicted expression preserves the spatial characteristics of biologically meaningful marker genes, we compute the Moran's I of seven representative DLPFC cortical layer markers on each of the 12 leave-one-out held-out slides, using a k-nearest-neighbour (k=6) spatial weight matrix in physical pixel coordinates. Table D.20 reports the mean Moran's I of the SpatialLIBD ground truth alongside the mean-of-20-sample predictions from Stem, STFlow, and PSSD. Both baselines produce Moran's I values that are systematically higher than the ground truth, which reflects a well-known artefact of averaging multiple stochastic samples and, in the case of STFlow, the additional smoothing induced by its local spatial attention. Across all seven markers, PSSD's Moran's I is the closest to the ground truth, with a mean absolute deviation of 0.082, compared to 0.239 for Stem and 0.264 for STFlow. Figure D.5 shows the same trend on layer-stratified violin plots: the spread of PSSD's per-spot predictions closely tracks the ground-truth distribution across cortical layers, whereas STFlow produces slightly narrower distributions consistent with mild spatial smoothing. Together, these results indicate that the progressive spatial-semantic decoupling in PSSD preserves biologically meaningful spatial heterogeneity rather than trading it away for spot-level accuracy.

Table D.11. Quantitative comparison of PSSD and baseline methods on the DLPFC, BC, and cSCC datasets. Reported values are Structural Similarity Index (SSIM↑) for the top 50 HVGs and top 50 HEGs. The best results are in **bold**; the second-best results are underlined.

Datasets	DLPFC		BC		cSCC	
Methods	HVG	HEG	HVG	HEG	HVG	HEG
ST-Net (He et al., 2020)	0.412 ± 0.0552	0.395 ± 0.0498	0.485 ± 0.0421	0.480 ± 0.0433	0.398 ± 0.0868	0.462 ± 0.1078
BLEEP (Xie et al., 2023)	0.438 ± 0.0587	0.428 ± 0.0534	0.510 ± 0.0445	0.503 ± 0.0451	0.435 ± 0.0919	0.485 ± 0.1132
Stem (Zhu et al., 2025)	0.466 ± 0.0594	0.457 ± 0.0562	0.545 ± 0.0472	0.540 ± 0.0480	0.461 ± 0.0930	0.521 ± 0.1212
STFlow (Huang et al., 2025)	0.478 ± 0.0603	0.470 ± 0.0575	0.556 ± 0.0480	0.552 ± 0.0487	0.480 ± 0.0952	0.535 ± 0.1245
PSSD (Ours)	**0.492 ± 0.0512**	**0.485 ± 0.0483**	**0.572 ± 0.0410**	**0.568 ± 0.0420**	**0.502 ± 0.0812**	**0.552 ± 0.1057**

Table D.12. Quantitative comparison of PSSD and baseline methods on three additional HEST-1k datasets. Reported values are SSIM↑ for the top 50 HVGs and top 50 HEGs. The best results are in **bold**; the second-best results are underlined.

Datasets	ccRCC		PAAD		COAD	
Methods	HVG	HEG	HVG	HEG	HVG	HEG
ST-Net (He et al., 2020)	0.553 ± 0.0890	0.836 ± 0.0561	0.479 ± 0.0364	0.934 ± 0.0164	0.645 ± 0.1973	0.762 ± 0.1683
BLEEP (Xie et al., 2023)	0.523 ± 0.0917	0.813 ± 0.0609	0.472 ± 0.0385	0.930 ± 0.0196	0.640 ± 0.1971	0.766 ± 0.1777
Stem (Zhu et al., 2025)	0.557 ± 0.0860	0.856 ± 0.0579	0.474 ± 0.0347	0.931 ± 0.0193	0.652 ± 0.1920	0.778 ± 0.1720
STFlow (Huang et al., 2025)	0.568 ± 0.1038	**0.865 ± 0.0690**	0.484 ± 0.0452	0.941 ± 0.0530	0.663 ± 0.1240	0.775 ± 0.1108
PSSD (Ours)	**0.570 ± 0.0812**	0.862 ± 0.0500	**0.491 ± 0.0325**	**0.945 ± 0.0146**	**0.665 ± 0.1780**	**0.783 ± 0.1510**

Table D.13. Quantitative comparison of PSSD and baseline methods on the Visium HD COAD dataset. Reported values are SSIM↑ for the top 50 HVGs and top 50 HEGs.

Methods	HVG	HEG
ST-Net (He et al., 2020)	0.027 ± 0.0029	0.024 ± 0.0018
BLEEP (Xie et al., 2023)	0.025 ± 0.0078	0.024 ± 0.0010
Stem (Zhu et al., 2025)	0.022 ± 0.0014	0.020 ± 0.0055
STFlow (Huang et al., 2025)	0.036 ± 0.0043	0.023 ± 0.0024
PSSD (Ours)	**0.041 ± 0.0065**	**0.027 ± 0.0009**

E. Additional Statistics

HVG/HEG gene set overlap To assess whether the HVG-based and HEG-based evaluations represent distinct tests of model performance, Table E.21 reports the intersection size and Jaccard index between the top 50 HVG and top 50 HEG sets across all seven datasets. On the original three benchmarks (DLPFC, BC, and cSCC) as well as on ccRCC and PAAD, the two sets overlap in at most 12 out of 50 genes (Jaccard ≤ 0.136), confirming that the HVG and HEG evaluations query largely disjoint sets of biologically informative genes. Higher overlap is observed on COAD (23 genes, Jaccard 0.299) and Visium HD COAD (32 genes, Jaccard 0.471), which reflects tissue-specific biology: in colorectal tissue, and especially in the low-transcript regime of Visium HD, a small pool of highly expressed genes also carries most of the between-spot variance. Given that the two gene sets are largely independent for most datasets, we report standard PCC on each set rather than the finer partitioning into overlapping, HVG-only, and HEG-only subsets, which would only be well defined for the two datasets with substantial overlap.

Runtime comparison Table E.22 summarizes the training and inference cost of PSSD and the four strongest baselines under each method's default training configuration on a single NVIDIA RTX 4090 GPU. Training cost is reported in GPU-hours per leave-one-out fold, and inference cost is reported in minutes per generated sample (per test slide) under each method's default sampling protocol, which is not identical across methods and is therefore made explicit in the "Sampling" column. ST-Net and BLEEP are the fastest because they are lightweight per-spot regressors that predict a single deterministic vector per spot in one forward pass, but this efficiency comes at the cost of visibly lower predictive fidelity in Table 1. STFlow uses a very short 5-step flow-matching schedule and reports a single per-spot prediction rather than a multi-sample average, so its inference is inexpensive but does not admit spot-level uncertainty. Stem and PSSD both invoke a 100-step integrator (DDPM for Stem, flow matching for PSSD) and average 20 samples per spot; this is the mechanism that gives them their heterogeneity-preserving behaviour (Figure 2, Table 5, Table D.20, and Figure D.5) and is used to produce the PCC/ARI/Moran's-I numbers reported throughout the paper. Under this shared 100-step 20-sample protocol, PSSD is roughly $8\times$ faster to sample than Stem on DLPFC while attaining higher PCC, ARI, and Moran's-I fidelity, indicating that its extra training cost is amortized by a substantially cheaper and more accurate sampling stage.

Table D.14. Quantitative comparison of PSSD and the two strongest generative baselines (Stem and STFlow) on the DLPFC, BC, and cSCC datasets using progressively larger gene sets. Reported values are PCC↑ for the top 50, top 100, and top 200 Highly Variable Genes (HVGs) and Highly Expressed Genes (HEGs). The best results are in **bold**; the second-best results are underlined.

Datasets	Methods	HVG			HEG		
		Top 50	Top 100	Top 200	Top 50	Top 100	Top 200
DLPFC	Stem (Zhu et al., 2025)	0.300 ± 0.0921	0.268 ± 0.1005	0.229 ± 0.1082	0.267 ± 0.0745	0.239 ± 0.0828	0.204 ± 0.0905
	STFlow (Huang et al., 2025)	0.313 ± 0.0872	0.286 ± 0.0951	0.251 ± 0.1028	0.279 ± 0.0795	0.257 ± 0.0867	0.226 ± 0.0942
	PSSD (Ours)	**0.337 ± 0.0770**	**0.319 ± 0.0812**	**0.295 ± 0.0866**	**0.299 ± 0.0673**	**0.283 ± 0.0715**	**0.263 ± 0.0769**
BC	Stem (Zhu et al., 2025)	0.348 ± 0.0578	0.316 ± 0.0654	0.283 ± 0.0740	0.351 ± 0.0549	0.318 ± 0.0624	0.287 ± 0.0708
	STFlow (Huang et al., 2025)	0.361 ± 0.0647	0.338 ± 0.0710	0.309 ± 0.0788	0.358 ± 0.0610	0.335 ± 0.0672	0.307 ± 0.0750
	PSSD (Ours)	**0.369 ± 0.0613**	**0.354 ± 0.0658**	**0.334 ± 0.0712**	**0.370 ± 0.0591**	**0.354 ± 0.0638**	**0.334 ± 0.0695**
cSCC	Stem (Zhu et al., 2025)	0.368 ± 0.2099	0.320 ± 0.2175	0.275 ± 0.2260	0.362 ± 0.2112	0.316 ± 0.2189	0.271 ± 0.2270
	STFlow (Huang et al., 2025)	0.383 ± 0.1975	0.346 ± 0.2036	0.305 ± 0.2118	0.390 ± 0.1937	0.354 ± 0.2000	0.313 ± 0.2080
	PSSD (Ours)	**0.416 ± 0.1837**	**0.390 ± 0.1888**	**0.360 ± 0.1958**	**0.411 ± 0.1856**	**0.386 ± 0.1910**	**0.356 ± 0.1982**

Table D.15. Results of ablation study on choice of pathology foundation model. Reported values are PCC↑ and MSE↓ for the top 50 HVGs and top 50 HEGs.

	HVG		HEG	
Foundation Model	PCC	MSE	PCC	MSE
CONCH (Lu et al., 2024)	0.354 ± 0.0592	0.197 ± 0.0932	0.358 ± 0.0720	0.212 ± 0.1059
UNI (Chen et al., 2024)	0.363 ± 0.0479	0.193 ± 0.1075	0.366 ± 0.0543	0.198 ± 0.0981
GigaPath (Xu et al., 2024)	0.357 ± 0.0602	0.191 ± 0.0953	0.365 ± 0.0627	0.201 ± 0.1105
CONCH + UNI	**0.369 ± 0.0613**	**0.188 ± 0.1021**	**0.370 ± 0.0591**	**0.195 ± 0.1057**

Table D.16. Ablation studies on the choice of ODE solver by the measurement of the average PCC for the top 50 HVGs and the average generation time per sample.

	HVG PCC			Sample time (minutes)		
ODE solver	DLPFC	BC	cSCC	DLPFC	BC	cSCC
Euler	**0.337**	**0.369**	0.416	**3.98**	**0.34**	**0.77**
RK4	**0.337**	0.368	0.414	13.81	1.16	2.75
Midpoint	0.334	0.367	0.416	7.43	0.62	1.49
Heun	0.335	0.367	**0.417**	6.87	0.58	1.40

Table D.17. Ablation studies on the number of gene neighbors by the measurement of the average PCC for the top 50 HVGs and top 50 HEGs.

Datasets	DLPFC		BC		cSCC	
Gene neighbors	HVG	HEG	HVG	HEG	HVG	HEG
4	0.323	0.293	0.361	0.363	0.402	0.400
6	0.331	0.297	0.365	0.361	0.406	0.406
8	0.336	**0.302**	0.364	0.367	0.413	0.410
**10**	**0.337**	0.299	**0.369**	0.370	**0.416**	**0.411**
12	0.335	0.301	0.367	**0.371**	0.415	0.407

Table D.18. Robustness to coordinate perturbation.

Noise (pixels)	0	5	10	20
Average λ	0.429	0.425	0.413	0.371
PCC(HVG)	0.337	0.334	0.328	0.315

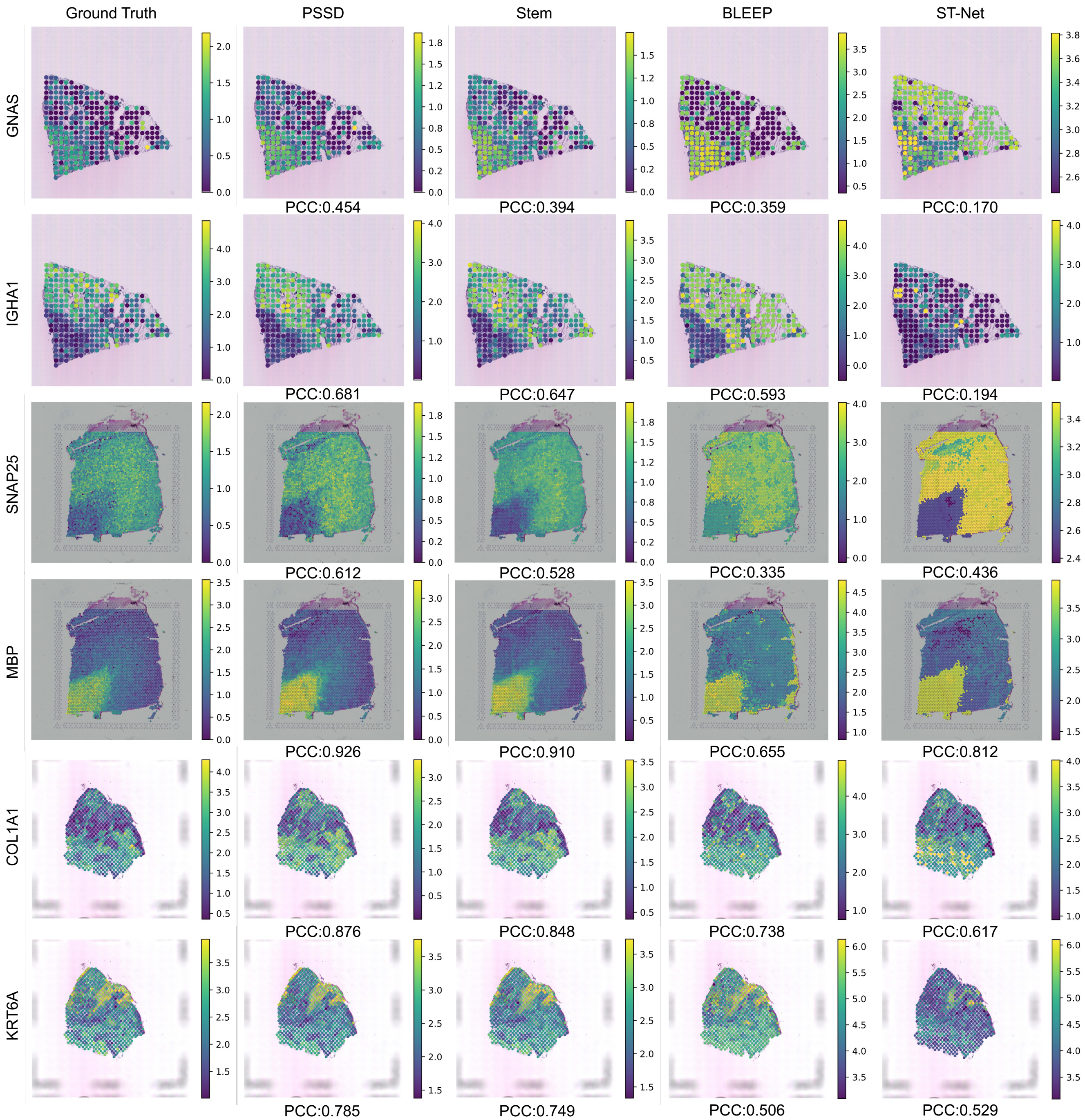

Figure D.3 The qualitative comparison includes the ground truth and predicted biomarker gene expression levels generated by PSSD, Stem, BLEEP, and ST-Net across samples from BC, DLPFC, and cSCC datasets.

Table D.19. Per-slide downstream tissue domain identification on the DLPFC dataset. For each of the 12 leave-one-out held-out slides, we cluster the predicted top 50 HVG expression with K-means ($K = 7$) after PCA reduction and evaluate against the SpatialLIBD manual cortical layer annotation using Adjusted Rand Index (ARI↑) and Normalised Mutual Information (NMI↑). The last row reports the mean ± std across the 12 slides. Per-row best values are in **bold**; the second-best are underlined.

Slide	Stem (Zhu et al., 2025)		STFlow (Huang et al., 2025)		PSSD (Ours)	
	ARI	NMI	ARI	NMI	ARI	NMI
MISC1	0.249	0.416	0.144	0.262	**0.300**	**0.433**
MISC2	0.256	0.407	0.151	0.268	**0.280**	**0.446**
MISC3	0.263	0.423	0.267	0.393	**0.283**	**0.426**
MISC4	0.094	0.229	0.152	0.282	**0.175**	**0.298**
MISC5	0.203	0.346	0.054	0.137	**0.292**	**0.431**
MISC6	0.194	0.329	0.068	0.180	**0.270**	**0.385**
MISC7	0.072	0.215	0.060	0.090	**0.147**	**0.336**
MISC8	0.021	0.129	0.075	0.121	**0.152**	**0.300**
MISC9	0.173	0.336	0.200	0.324	**0.219**	**0.381**
MISC10	0.224	0.358	0.211	0.328	**0.245**	**0.418**
MISC11	0.255	0.401	0.197	0.346	**0.291**	**0.429**
MISC12	0.188	0.295	0.136	0.230	**0.247**	**0.392**
Mean ± std	0.183 ± 0.077	0.324 ± 0.088	0.143 ± 0.065	0.247 ± 0.093	**0.242 ± 0.054**	**0.390 ± 0.050**

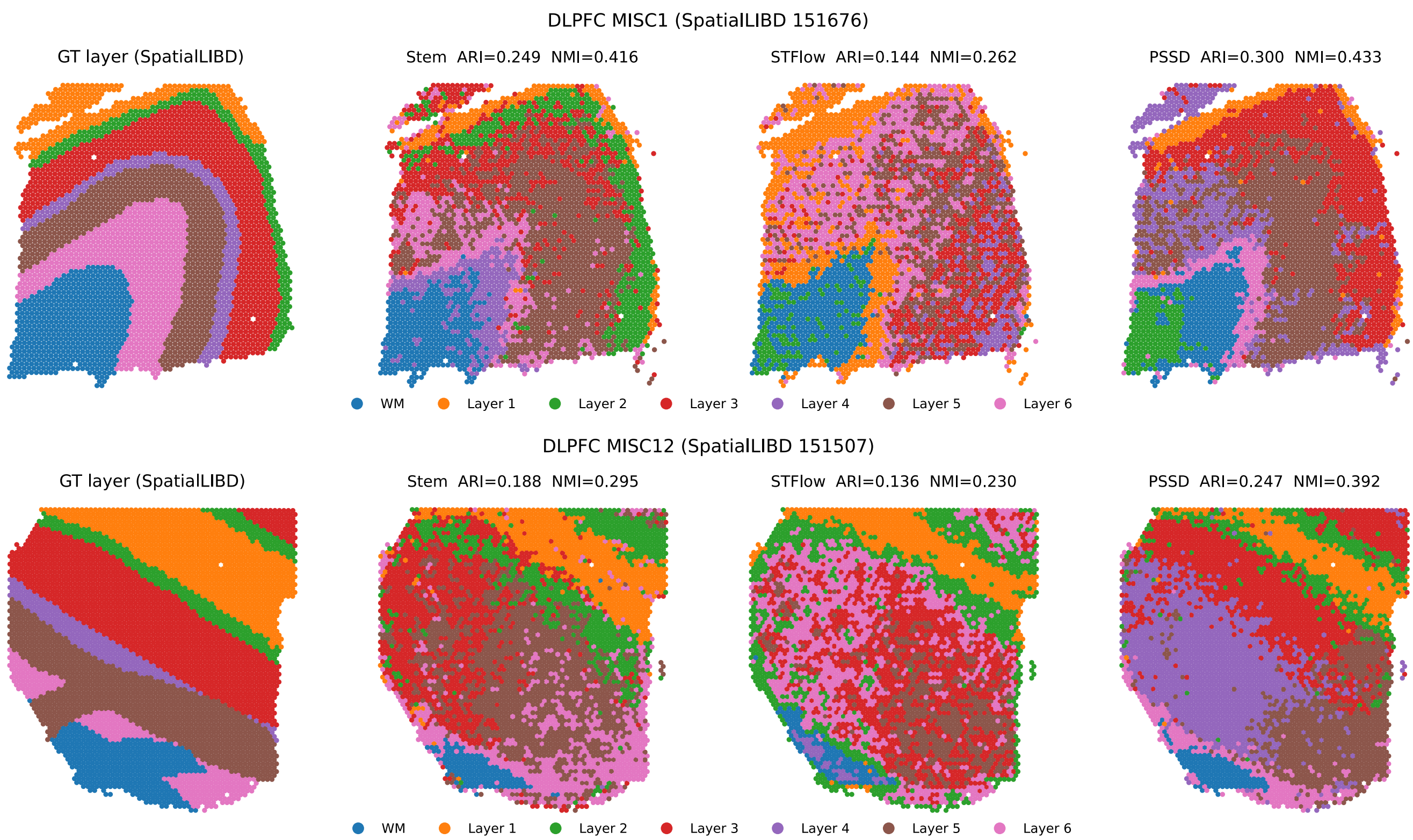

Figure D.4 Spatial domain identification on two representative DLPFC slides (MISC1 and MISC12). Each row shows the SpatialLIBD ground-truth cortical layer annotation and the K-means clustering of the top 50 HVG expression predicted by Stem, STFlow, and PSSD. ARI and NMI computed against the SpatialLIBD annotation are reported above each predicted panel.

Table D.20. Spatial autocorrelation (Moran's I) of representative DLPFC cortical layer markers on the SpatialLIBD ground truth and the mean-of-20-sample predictions from Stem, STFlow, and PSSD, averaged over the 12 leave-one-out slides. $|\Delta|$ is the absolute distance to the ground-truth Moran's I; smaller is better. The best (closest to GT) result per row is in **bold**; the second-best is underlined.

Marker	GT	Stem (Zhu et al., 2025)		STFlow (Huang et al., 2025)		PSSD (Ours)	
		Moran's I	$\|\Delta\|$	Moran's I	$\|\Delta\|$	Moran's I	$\|\Delta\|$
MBP	0.661	0.797	0.136	0.810	0.149	**0.735**	**0.074**
MOBP	0.394	0.671	0.277	0.690	0.296	**0.478**	**0.084**
PLP1	0.545	0.741	0.196	0.760	0.215	**0.628**	**0.083**
PCP4	0.195	0.400	0.205	0.445	0.250	**0.242**	**0.047**
SNAP25	0.310	0.598	0.288	0.628	0.318	**0.410**	**0.100**
CCK	0.198	0.456	0.258	0.485	0.287	**0.315**	**0.117**
CD74	0.127	0.436	0.309	0.460	0.333	**0.198**	**0.071**
Mean	0.347	0.586	0.239	0.611	0.264	**0.429**	**0.082**

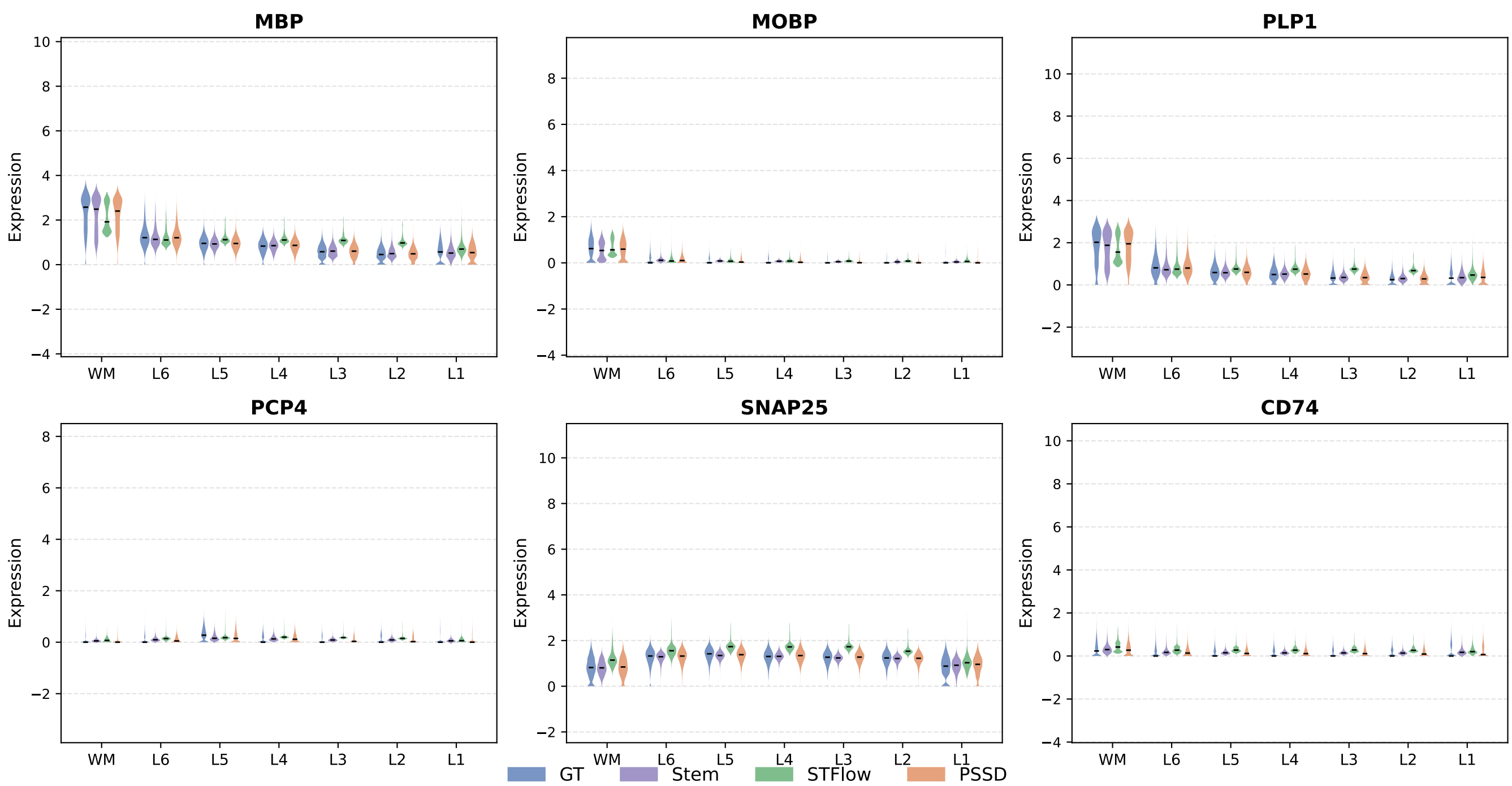

Figure D.5 Violin plots of representative DLPFC cortical layer markers stratified by SpatialLIBD layer annotation. For each marker (subplot), each layer shows side-by-side violins for the SpatialLIBD ground-truth expression and the mean-of-20-sample predictions from Stem, STFlow, and PSSD, pooled across the 12 leave-one-out held-out slides. Median is indicated by the black horizontal line inside each violin.

Table E.21. Overlap between the top 50 HVG and top 50 HEG gene sets for each dataset. |HVG∩HEG| counts the genes present in both sets; the Jaccard index normalises the intersection by the union of the two sets.

Dataset	|HVG ∩ HEG|	Jaccard
DLPFC	3	0.031
BC	7	0.075
cSCC	2	0.020
ccRCC	12	0.136
PAAD	3	0.031
COAD	23	0.299
Visium HD COAD	32	0.471

Table E.22. Training and inference cost of PSSD and the four strongest baselines under their default training configuration on a single NVIDIA RTX 4090 GPU. Training is reported in GPU-hours per leave-one-out fold; inference is reported in minutes per generated sample per test slide. The "Sampling" column shows each method's default per-slide sampling protocol as (integration steps) × (number of samples averaged per spot); entries marked "regression" or "retrieval" are single deterministic forward passes.

Methods	Sampling	**Training (h / fold)**			**Inference (min / sample)**		
		DLPFC	BC	cSCC	DLPFC	BC	cSCC
ST-Net (He et al., 2020)	regression	1.5	1.0	0.8	0.05	0.01	0.02
BLEEP (Xie et al., 2023)	retrieval	1.0	0.7	0.5	0.51	0.06	0.12
Stem (Zhu et al., 2025)	100 × 20	19.4	17.8	13.1	32.04	2.36	5.47
STFlow (Huang et al., 2025)	5 × 1	1.5	0.15	0.30	0.003	0.0002	0.0005
PSSD (Ours)	100 × 20	26.1	25.9	19.7	3.98	0.34	0.77
